# Tirap controls *Mycobacterium tuberculosis* phagosomal acidification

**DOI:** 10.1371/journal.ppat.1011192

**Published:** 2023-03-08

**Authors:** Imène Belhaouane, Amine Pochet, Jonathan Chatagnon, Eik Hoffmann, Christophe J. Queval, Nathalie Deboosère, Céline Boidin-Wichlacz, Laleh Majlessi, Valentin Sencio, Séverine Heumel, Alexandre Vandeputte, Elisabeth Werkmeister, Laurence Fievez, Fabrice Bureau, Yves Rouillé, François Trottein, Mathias Chamaillard, Priscille Brodin, Arnaud Machelart

**Affiliations:** 1 Univ. Lille, CNRS, INSERM, CHU Lille, Institut Pasteur de Lille, U1019—UMR 9017—CIIL—Center for Infection and Immunity of Lille, Lille, France; 2 High Throughput Screening Laboratory, The Francis Crick Institute, London, United Kingdom; 3 Pasteur-TheraVectys Joint Lab, Institut Pasteur, Université Paris Cité, Paris, France; 4 Univ. Lille, CNRS, Inserm, CHU Lille, Institut Pasteur de Lille, US 41—UMS 2014—PLBS, Lille, France; 5 Laboratory of Cellular and Molecular Immunology, GIGA-Research, Liège, Belgium; 6 Laboratory of Cell Physiology, INSERM U1003, University of Lille, Lille, France; McGill UniversityHealth Centre, CANADA

## Abstract

Progression of tuberculosis is tightly linked to a disordered immune balance, resulting in inability of the host to restrict intracellular bacterial replication and its subsequent dissemination. The immune response is mainly characterized by an orchestrated recruitment of inflammatory cells secreting cytokines. This response results from the activation of innate immunity receptors that trigger downstream intracellular signaling pathways involving adaptor proteins such as the TIR-containing adaptor protein (Tirap). In humans, resistance to tuberculosis is associated with a loss-of-function in Tirap. Here, we explore how genetic deficiency in Tirap impacts resistance to *Mycobacterium tuberculosis* (Mtb) infection in a mouse model and *ex vivo*. Interestingly, compared to wild type littermates, Tirap heterozygous mice were more resistant to Mtb infection. Upon investigation at the cellular level, we observed that mycobacteria were not able to replicate in Tirap-deficient macrophages compared to wild type counterparts. We next showed that Mtb infection induced Tirap expression which prevented phagosomal acidification and rupture. We further demonstrate that the Tirap-mediated anti-tuberculosis effect occurs through a Cish-dependent signaling pathway. Our findings provide new molecular evidence about how Mtb manipulates innate immune signaling to enable intracellular replication and survival of the pathogen, thus paving the way for host-directed approaches to treat tuberculosis.

## Author summary

The efficiency of *Mycobacterium tuberculosis* in establishing its replicative niche relies on its capacity to manipulate host factors. The aim of our study was to identify the impact of a genetic deficiency in Tirap, a key factor of the innate immunity response, on the evolution of *in vivo* and *ex vivo* tuberculosis infections. Here, we show that mice heterozygous for a truncated Tirap protein were more resistant to the infection, displaying less bacterial burden in the lungs. At the cellular level, we identified a repression of Cish-mediated phagosomal acidification in Tirap-deficient macrophages resulting in an enhanced bactericidal activity. Our results provide new insights into how Mtb infection promotes Tirap expression to ensure its survival.

## Introduction

In a given population, individuals differ from each other in many of their genes. Infectious diseases have been major threats to health and survival throughout the history of human evolution. Natural selection is thereby expected to act significantly on host defense genes, particularly on innate immunity genes, whose products mediate the host interaction with the microbial environment [1]. Toll like receptors (TLRs) play a central role in the coordination of innate and adaptive immunity. TLRs can recognize a distinct range of conserved microbial components. The TLR-mediated detection of microbes activates a signalling cascade that leads to the initiation of an immunoregulatory response. The variability of this immune response is genetically controlled. In humans, genetic deficits and polymorphisms associated with TLR pathways are often described as influencing the course of many infections [2–4].

Among the various immune actors involved in this process, the *tirap* gene, located on chromosome 11q24.2, encodes for the Tirap/Mal (TIR-containing adaptor protein/MyD88 adaptor-like) protein, which has a C-terminal TIR domain acting as a sorting and bridging adaptor between TLR2, TLR4 and TLR9 to bring the TLR adaptor MyD88 (myeloid differentiation primary response 88) into the immune pathway [5,6]. The signalling cascade initiated upon TLR stimulation leads to the activation of transcription factors resulting in the production of pro-inflammatory cytokines such as Interleukin (IL)-6, IL-12 and Tumour Necrosis Factor alpha (TNF-α) [7]. Tirap knock-out mice have been reported to be more susceptible to *Escherichia coli* [8] and *Klebsiella pneumonia* [9] infection. Furthermore, Tirap is the most polymorphic of all the adaptor proteins. In human populations, more than 30 SNPs (single nucleotide polymorphism) have been described in the Tirap protein and the surrounding region (8 of which are in its coding region) [10]. These genetic variations have been associated with both disease susceptibility and resistance, suggesting paradoxical functions of Tirap to contain infections [11].

Contrasting roles of Tirap genetic variations have been particularly reported in patients facing tuberculosis (TB) [12–15]. TB is a human infectious lung disease that kills more than 1.5 million people each year [16]. *Mycobacterium tuberculosis* (Mtb), the bacterium responsible for the disease, is a successful pathogen, which, upon inhalation, is able to infect host alveolar macrophages and other cell types. The bacillus will then massively manipulate endocytic trafficking of macrophages to ensure its survival [17]. One of these survival strategies is the manipulation of endosomal and lysosomal host compartments, which prevents the acidification of Mtb-containing vacuoles by blocking phagosome maturation [18]. Ultimately, Mtb can escape from the vacuole and reside inside the cell cytoplasm [19]. Therefore, Mtb has developed an extensive set of mechanisms to promote its survival and replicate intracellularly during its prolonged stay in the alveolar macrophages.

Epidemiological studies have identified a role of Tirap genetic variation and more precisely the S180L mutation in TB susceptibility [20–23]. Amino acid substitution of a serine by a leucine at position 180 leads to altered NF-κB signalling and lastly protection against exaggerated inflammation [10]. The gene may have this polymorphism as homozygous ser/ser or leu/leu or heterozygous as ser/leu motif. The S180L (C539T) has been reported to be protective against several diseases but its role in TB susceptibility has been largely debated. For example, Capparelli et al. demonstrated that S180L heterozygosity confers resistance against TB in individuals [22], whereas another study showed the completely opposite effect of S180L [21]. Even though a meta-analysis concluded that S180L polymorphism is significantly correlated with reduced risk of TB infection [15], further investigations are needed to understand the roles of this protein in the control of TB.

In this work, we compared the ability of wild type (WT/Tirap^+/+^), heterozygous (Tirap^+/-^) or homozygous knock-out (Tirap^-/-^) mice for Tirap to control Mtb infection. Interestingly, we observed that heterozygous mice are the more resistant to infection. At the infected macrophage level, we observed in Tirap KO cells that the bacterium was unable to control Cish-mediated intra-endocytic processes, corroborating with reduced Mtb replication. Together these results suggest that a partial loss-of-function in the Tirap protein protects against Mtb infection.

## Results

### *In vivo* model of Tirap heterozygosity protects from Mtb infection

**Fig 1:**
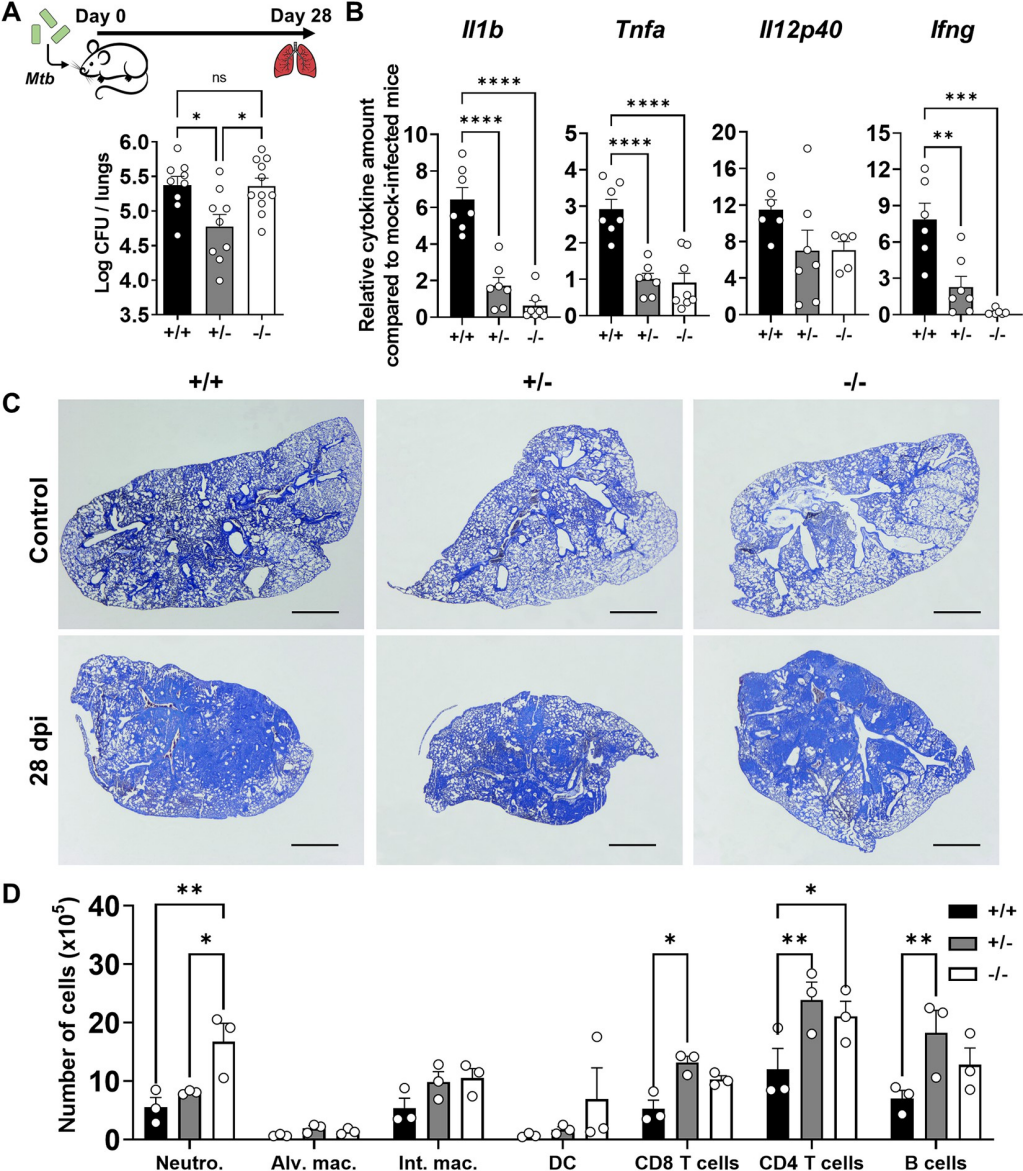
Comparative study of bacterial load, recruited immune cells and cytokine expression in lungs of infected Tirap +/+, +/- and -/- mice. C57BL/6 WT (+/+), Tirap^+/-^ (+/-) and Tirap^-/-^ (-/-) mice were intranasally infected with Mtb H37Rv (10^5^ CFU/20μL). 28 days post-infection, mice were sacrificed to study their pathological state. (**A**) Mycobacterial load was determined by plating lung lysates and counting Colony-Forming Units (CFU) 2 weeks after plating. Shown are mean ± SEM of at least 9 infected mice per condition of three independent experiments. (**B**) Histograms showing relative cytokine amounts in lung lysates of infected +/+, +/- and -/- mice compared to mock-infected mice. Shown are mean ± SEM of 7 infected mice per condition of one representative out of two independent experiments. (**C**) Toluidine blue staining of non-infected (control) and infected (28 days post-infection) slice lungs (5 μm) from WT, Tirap^+/-^ and Tirap^-/-^ mice. Bar: 10 mm. (**D**) Histogram depicting changes of immune cells populations during Mtb H37Rv infection, as determined by flow cytometry using specific cell surface markers. Cell numbers were normalized to the total cell number analyzed in the whole lung. Shown are mean ± SEM of cells obtained from three individual mice per group representative of two independent experiments. dpi: days post-infection, ns: non-significant, * P value < 0.05, ** P value < 0.01, as determined by one-way ANOVA test.

C57BL/6 Tirap-deficient mice (Tirap^-/-^) have been historically generated by gene targeting leading to the depletion of two exons coding for the TIR domain and the expression of non-functional Tirap protein [24]. In accordance with the objective of this work, we generated a mouse model for Tirap heterozygosity (Tirap^+/-^) by cross-breeding wild type (Tirap^+/+^) and knock-out (Tirap^-/-^) mice (**S1 Fig**). To determine the physiological relevance of a total or a partial loss of Tirap function during TB infection, we inoculated intranasally these mice with a high dose (1 x 10^5^ colony-forming units (CFU)) of the virulent Mtb H37Rv strain. During the experiment, no apparent differences in body weight or animal behavior were observed. 28 days post-infection, mice were sacrificed and lungs were harvested for histopathology, immunopathology and to study bacterial burden. Interestingly, determination of pulmonary mycobacterial loads showed a significant increase in Tirap^+/+^ and Tirap^-/-^ mice compared to Tirap^+/-^ littermates suggesting that the latter are more resistant to infection (**Fig 1A**). To gain insight into the pathogenesis established during infection, we analyzed lung sections, cytokine production and immune cell recruitment to the lungs.

First, the inflammatory cytokine profiles in lungs of infected Tirap^+/-^ and Tirap^-/-^ mice show a significant decrease in the amount of TNFα, IL1β and IFNγ compared to wild type animals (**Fig 1B**). A decrease in the transcription of IL12p40 and TNFα was also detected in lungs of Tirap-deficient mice relative to wild type mice (**S2 Fig**). Even if anatomopathological examination between the different Tirap genotypes revealed no difference in the overall aspect of the lungs and the lesions induced by the infection (**Fig 1C**), we compared the recruitment of immune cells by flow cytometry. While the overall basal number of immune cells present in the lungs of each genotype was not significantly different (**S1 Fig**), the examination of innate immune cell profiles 28 days post-infection revealed a noticeable increase in neutrophils in the lungs of Tirap^-/-^ mice (**Fig 1D**). In contrast, the number of CD8 T cells, CD4 T cells and B lymphocytes increased in lungs of infected Tirap^+/-^ mice, but not in Tirap^+/+^ and Tirap^-/-^ mice, suggesting a more effective adaptive immune response, which could explain the resistance of heterozygous mice to Mtb infection (**Fig 1D**).

Taken together, our results show that Tirap^+/-^ mice exhibit more pronounced control of Mtb infection. These observations are consistent with scientific evidence in human showing that Tirap SNP heterozygosity is associated with protection. Given the observations at the tissue level, we went on to study Mtb replication *in cellulo* to determine the role of Tirap at the host cell level.

### Tirap deficiency impairs Mtb replication inside macrophages

**Fig 2:**
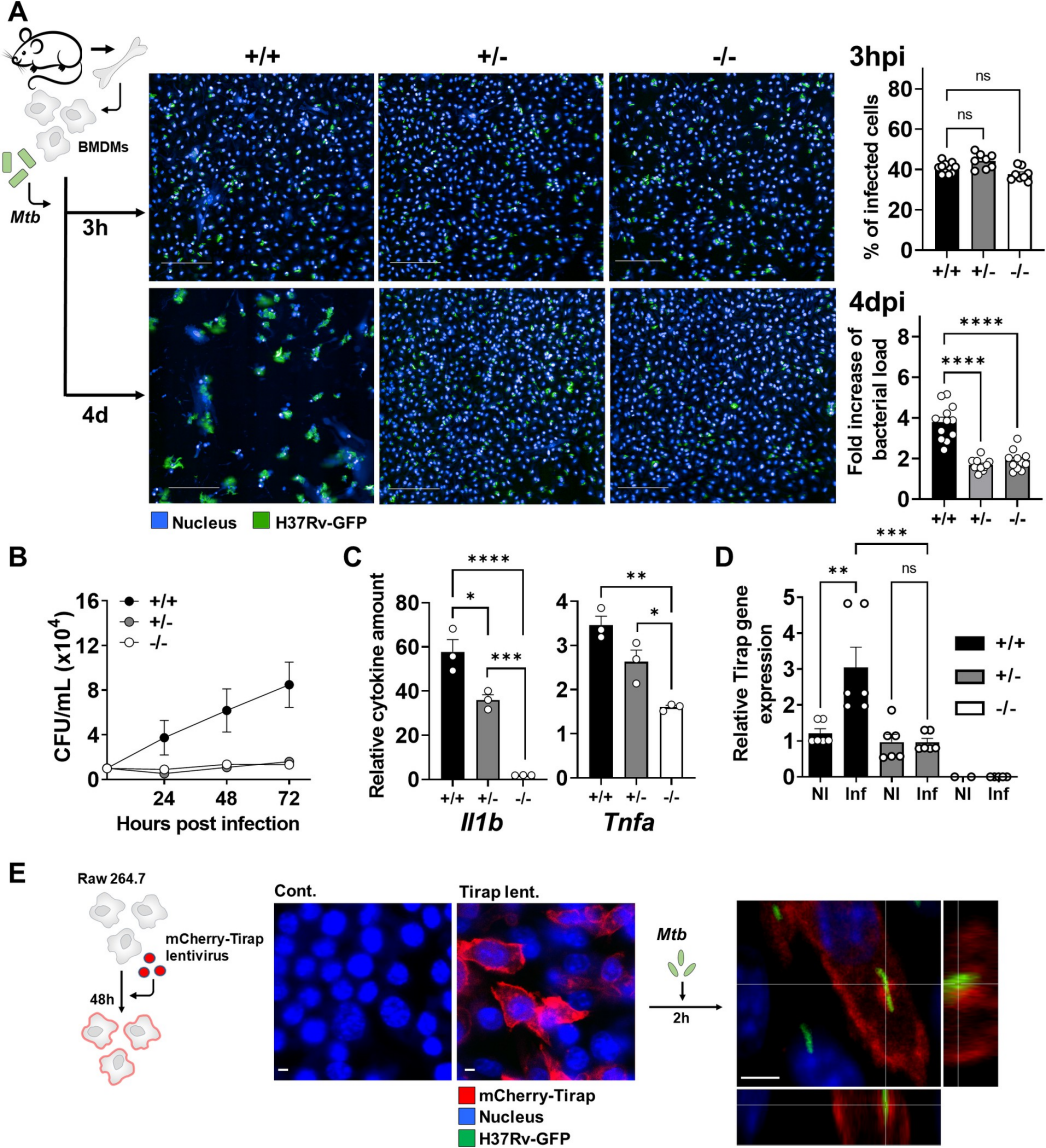
Tirap expression and localization in Mtb-infected BMDMs: Impact on bacterial growth and cytokine expression. (**A**) BMDMs were grown in 384-well plates, infected with Mtb H37Rv-GFP (MOI of 2) and analyzed by automated confocal microscopy. Shown are representative images of Tirap^+/+^, Tirap^+/-^ and Tirap^-/-^ BMDMs at 3 hpi (upper panel) and 4 dpi (lower panel). Segmentation algorithms were applied to input images to detect nuclei labeled by Hoechst 33342 (cyan) and the GFP signal of Mtb H37Rv (green) to determine infection rate 3 hpi (upper histogram) and replication fold increase from 3 hpi to 4 dpi (lower histogram). Shown are mean ± SEM of at least 8 analyzed wells per condition of one representative out of three independent experiments. Bar: 50 μm. (**B**) BMDMs were grown in 24-well plates, infected with Mtb H37Rv (MOI of 2) and lysed at 2, 24, 48 and 72 hpi. Serial dilutions of lysates were plated out to determine bacterial load by counting CFUs. Shown are mean ± SEM of 4 wells per condition of one representative out of two independent experiments. (**C**) Histograms showing relative cytokine amounts in supernatant of infected WT, Tirap^+/-^ and Tirap^-/-^ BMDMs 96 hpi compared to non-infected cells. Shown are mean ± SEM of three infected wells. (**D**) Histogram showing mean ± SEM of relative *Tirap* gene expression. WT, Tirap^+/-^ and Tirap^-/-^ BMDMs were infected with Mtb H37Rv at MOI of 2 for 3 h. Non-infected (NI) WT cells served as control. Transcription of *Tirap* was assessed by quantitative RT-PCR and normalized to the expression of *Gapdh*, used in all samples as a housekeeping gene. (**E**) RAW264.7 cells were transfected with mCherry-Tirap vector expressing lentivirus for 48 h prior to infection with Mtb H37Rv-GFP. Confocal microscopy imaging shows subcellular localization of Tirap (m-cherry) and Mtb H37Rv-GFP (green). Bar: 5 μm. NI: non-infected, Inf: infected, hpi: hours post-infection, dpi: days post-infection, ns: non-significant, * P value < 0.05, ** P value < 0.01, *** P value < 0.001, **** P value < 0.0001, as determined by one-way ANOVA test.

To evaluate the contribution of Tirap within the main intracellular niche of Mtb, we first assessed its effect on Mtb replication in primary murine macrophages. We first ensured that the proliferative capacity of the BMDMs was not altered by Tirap deficiency. As shown in **S1 Fig**, the three types of macrophages exhibited the same growth kinetics over a period of 4 days. Bone marrow-derived macrophages (BMDMs) derived from each genotype of mice were then infected with a GFP-expressing Mtb H37Rv strain (MOI (multiplicity of infection) of 2) and intracellular bacterial growth was quantified at day 4 post-infection. Cells were grown in 384-well plates and their nuclei were labeled enabling analysis by an automated confocal microscopy approach using in-house multiparametric image analysis. This allowed acquisition and examination of hundreds of images generating robust and reproducible data sets [25]. We applied segmentation algorithms to input images, allowing us to distinguish nuclei and intracellular bacteria and to determine the percentage of infected cells and the intracellular bacterial load per cell. Tirap deficiency did not impair Mtb uptake by macrophages, as the percentages of Mtb-infected BMDMs 3 h post-infection (hpi) were similar (**Fig 2A**). We then compared the intracellular area of Mtb per infected cell, which directly correlates with the bacterial load [18]. At 4 days post-infection (dpi), increase of bacterial load was lower in Tirap^+/-^ BMDMs than Tirap^+/+^ BMDMs, which is consistent with the phenotype observed in mice (**Fig 2A**). Intriguingly, fully deficient BMDMs also showed a lower bacterial load than Tirap^+/+^ BMDMs and a similar load compared to that of Tirap^+/-^ BMDMs. The phenotype was confirmed by CFU plating of infected macrophages (**Fig 2B**). Briefly, while only heterozygous mice are associated with resistance, both heterozygous and homozygous Tirap-deficient macrophages prevented Mtb replication.

We next compared the inflammatory response initiated after BMDM infection and observed that, similarly to *in vivo* results, TNFα and IL1β production was lower in Tirap^+/-^ and Tirap^-/-^ macrophages compared to infected WT cells (**Fig 2C**). Therefore, at the gene expression level, *Tnf-α*, *Il1β*, *Il6*, *Il12p40* and *Nos2* were significantly lower in deficient cells compared to the WT (**S2 Fig**). Surprisingly in our model, a lower inflammatory response is associated with resistance to infection. The contradictory phenotype observed between Tirap^-/-^ mice and macrophages could be explained by the recruitment of neutrophils in the mouse tissue, which would compensate bacterial replication.

We then sought to understand why Tirap promotes Mtb replication inside macrophages. First, we measured Tirap expression in infected BMDMs by quantitative RT-PCR. While *Tirap* was expressed at steady state in non-infected (NI) Tirap^+/+^ and Tirap^+/-^ BMDMs at a similar rate, we observed a pronounced difference during *Mtb* infection at 3 hpi (MOI of 2). Indeed, *Tirap* expression increased by three-fold in Tirap^+/+^ BMDMs upon infection (**Fig 2D**). Moreover, no induction of the transcription of *Tirap* was observed in heterozygous cells upon Mtb infection. As expected, Tirap^-/-^ macrophages lacked expression of Tirap. This suggests that bacteria engage Tirap and/or its downstream signaling pathways to replicate inside macrophages.

Second, we aimed at analyzing the cellular distribution of Tirap. To this end, murine RAW264.7 macrophages were transfected with a fluorescently-coupled mCherry-Tirap lentiviral vector prior to infection with Mtb H37Rv-GFP (**Fig 2E**). Using confocal microscopy, we found that the Tirap signal localized close to bacteria, indicating that the adaptor might be recruited to the Mtb-containing vacuole (MCV) during infection.

Taken together, these data suggest that the increased expression of Tirap and its recruitment to the MCV during infection is the first evidence that Mtb has the capacity to dampen host defense mechanisms *via* Tirap. Moreover, our results align with the intracellular replication of Mtb and the activation of inflammatory responses. To investigate this further, we compared the intracellular trafficking pathways of Mtb in different types of macrophages.

### Tirap prevents acidification of the Mtb-containing vacuole (MCV) allowing bacteria to reach their replicative niche

**Fig 3:**
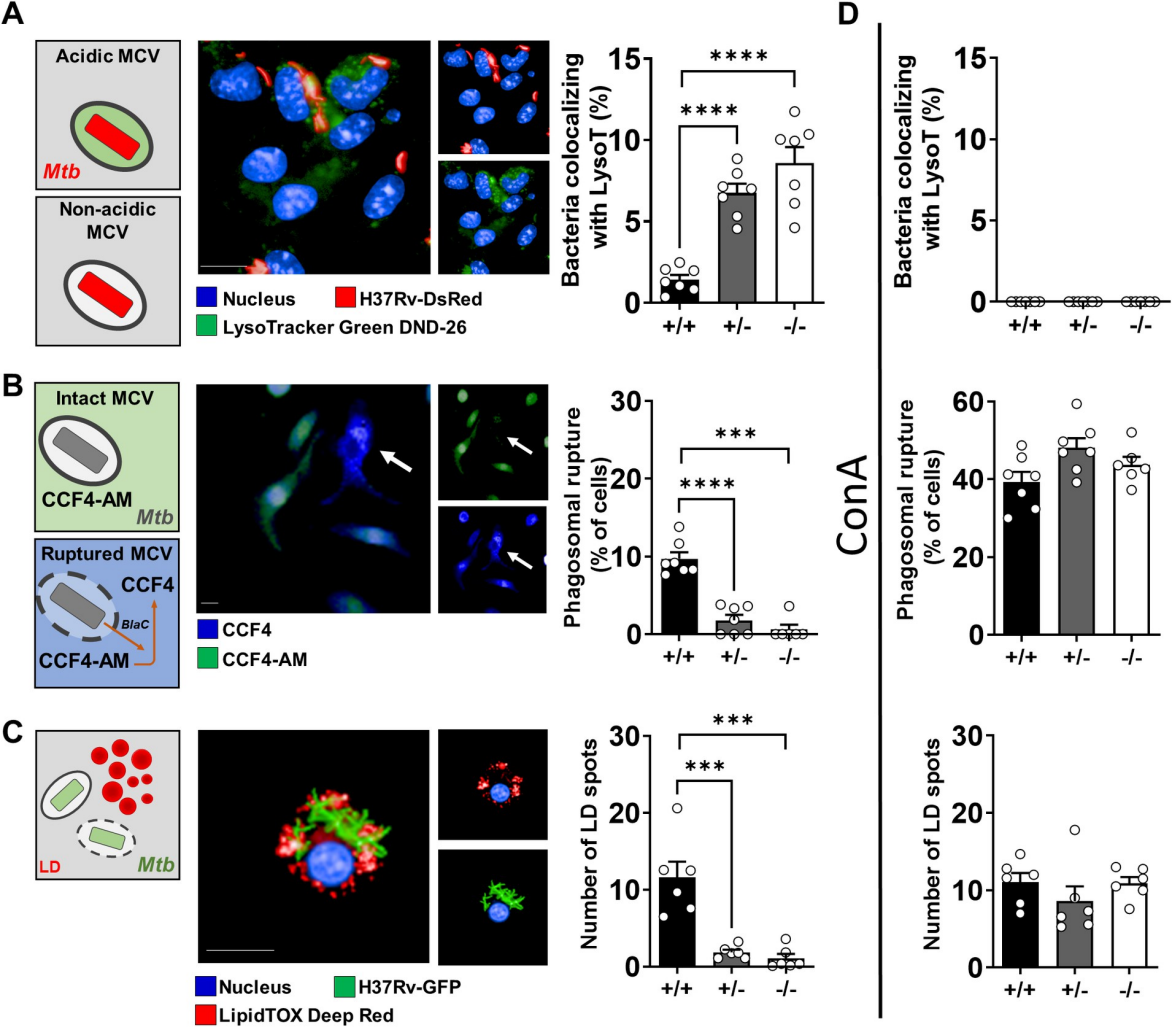
Tirap implication in phagosomal maturation and lipid droplet formation. Typical images and related quantifications (representative of two independent experiments) of phagosome acidification (**A**), phagosome rupture (**B**) and lipid droplet formation (**C**) of Mtb H37Rv infected BMDMs treated (**D**) or not with ConA. (**A**) DAPI-labelled cell nuclei are shown in blue, Mtb H37Rv-DsRed in red and acidic-pH-sensitive LysoTracker staining in green. The LysoTracker signal was set to the minimum in non-infected controls. Bar: 10 μm. Histograms showing mean ± SEM obtained from 7 wells of the percentage of bacteria displaying a Lysotracker signal within the cells. (**B**) Representative images showing cells displaying CCF4-AM staining (green) or CCF4 staining (blue) corresponding to phagosomal rupture. Histograms showing mean ± SEM obtained from 7 wells of the percentage of BMDMs detected with phagosomal rupture 24 hpi. Bar: 5 μm. (**C**) Representative images showing LD in Mtb infected cells. DAPI-labelled cell nuclei are in blue, Mtb H37Rv-GFP is in green, and LD staining (LipidTox) is in red. Bar: 5 μm. Histogram showing the average number ± SEM of LD per cell at 96 hpi obtained from at least 6 wells. LD: lipid droplets, * P value < 0.05, ** P value < 0.01, *** P value < 0.001, **** P value < 0.0001, as determined by one-way ANOVA test.

To investigate phagosomal maturation, we first monitored the fusion of MCVs with lysosomes by fluorescence microscopy using the pH-sensitive LysoTracker dye. It has been previously demonstrated that the intensity of LysoTracker labeling directly correlates with the acidification of Mtb vacuoles [18]. Upon Mtb infection, LysoTracker intensity was significantly higher in Tirap^+/-^ and Tirap^-/-^ cells than in WT cells (**Fig 3A**). These results suggest that Tirap is involved in the blockade of phagosomal acidification that is required for Mtb replication.

Although several studies have demonstrated that MCVs remain features of endosomes and early phagolysosomes (for comprehensive review [26]), others reported that the phagosome membrane rapidly undergoes progressive disruption through the action of the canonical bacterial virulence factors phthiocerol dimycocerosate (PDIM) and ESX-1 [27,28]. This phagosomal permeabilization allows Mtb to access cytosolic components that may contribute to mycobacterial replication and to induce cell necrosis for further dissemination [19,29]. This led us to assess whether Tirap deficiency could interfere with Mtb-induced phagosomal rupture. We used the FRET CCF4-AM dye, a cell permeable β-lactamase substrate that shifts its emission wavelength from green to blue when converted into its negatively charged metabolite CCF4 [30]. As Mtb expresses membrane-associated β-lactamase BlaC, the green-to-blue ratio of the dye has been often used as a surrogate marker correlating with the ability of mycobacteria to escape their phagosomal vacuoles and to reach the cytosol [19,31]. At 24 hpi, the percentage of Mtb phagosomal rupture in Tirap^+/-^ and Tirap^-/-^ infected BMDMs was lower than in Tirap^+/+^ BMDMs, correlating with the increased acidification in Tirap-deficient cells compared to WT cells (**Fig 3B**). It is also established that Mtb persistence and replication rely on host metabolic homeostasis shift and availability of host nutrients in the cytosol. Among the latter, lipid droplets (LD) are a key reservoir of neutral host lipids that are important carbon sources required during Mtb replication [32]. Whereas large amounts of LD were present upon Mtb infection at 4 dpi in WT BMDMs, the number of LD spots in Mtb-infected Tirap-deficient cells (both heterozygous and homozygous) was almost absent (**Fig 3C**). This suggests that in Tirap-expressing cells, Mtb has likely more access to host nutrients that fuel intracellular replication than in Tirap-deficient cells.

Taken together, these observations showed that in WT macrophages, Tirap impacts Mtb trafficking thereby promoting bacterial replication. Indeed, Tirap is required for the control of phagosomal acidification that is necessary for phagosomal escape, intracellular replication and reprogramming of host metabolism. To determine whether there is a direct link between phagosome acidification, phagosomal rupture and LD formation, we followed Mtb intracellular trafficking in macrophages treated with the phagosomal acidification inhibitor concanamycin A (conA). ConA specifically inhibits vacuolar type H+-ATPase and thereby prevents the functions of acidic organelles [33]. Interestingly, the blockade of vacuolar acidification in Tirap-deficient macrophages results in a phenotype similar to that of WT with increased Mtb phagosomal escape and a higher number of LD in infected cells (**Fig 3D**). These results support that control of phagosomal acidification is dependent on a Tirap-mediated pathway.

### Tirap induces Cish signalling pathway

**Fig 4:**
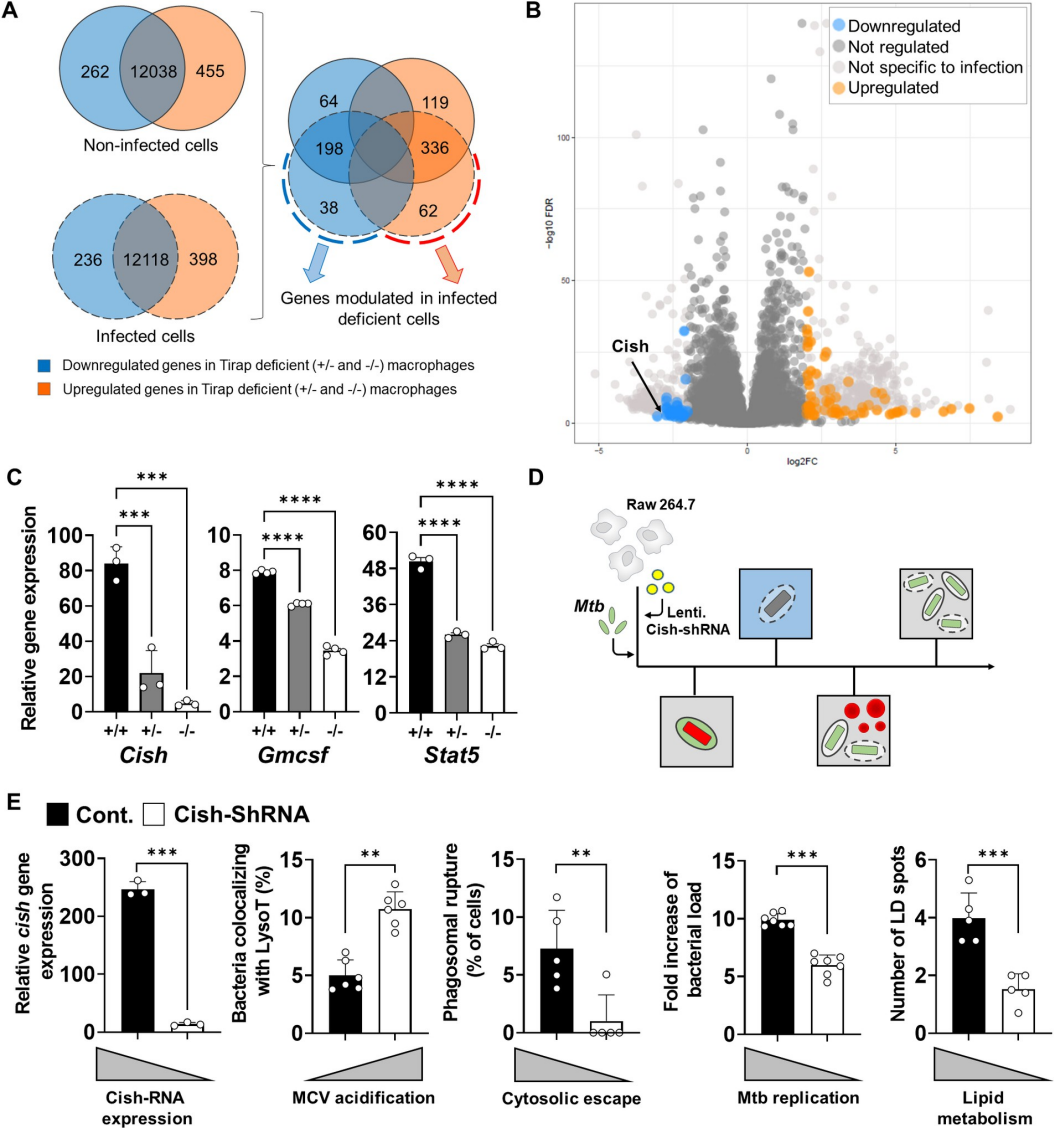
Identification of differentially regulated genes between +/+, +/- and -/- BMDMs during Mtb infection. (**A**) Total RNA was extracted from non-infected and infected (3 h) WT, Tirap^+/-^ and Tirap^-/-^ BMDMs and sequenced with the Illumina system and Deseq2 analysis as described in the Materials and Methods section. (**B**) Volcano plot of RNAseq data from non-infected versus infected cells showing the adjusted p-value (false discovery rate, FDR -log10) versus fold change, FC (log2). The 100 genes with an FDR < 0.01 and FC > 2 are shown in blue and orange for downregulated and upregulated genes, respectively. (**C**) Histograms showing mean ± SEM of gene expression levels in BMDMs from WT, Tirap^+/-^ and Tirap^-/-^ mice 3 hpi. *Cish* and *Gmcsf* expression was quantified by quantitative RT-PCR. *Stat5* expression level is reported from RNAseq data. (**D**) Schema of the different subcellular events studied in infected RAW264.7 cells silenced for *Cish* gene expression. (**E**) RAW264.7 cells were grown in 384-well plates, infected with Mtb H37Rv (MOI of 2) and analyzed by automated confocal microscopy. Histograms show the quantification of *Cish* gene expression and different phenotypic subcellular events during Mtb infection of WT (Cont) and silenced (Cish-shRNA) RAW264.7 cells. ** P value < 0.01, *** P value < 0.001, **** P value < 0.0001, as determined by one-way ANOVA test.

To better understand how a lack of Tirap results in significant containment of Mtb growth in macrophages, we compared the expression profiles of Mtb-infected WT and Tirap-deficient BMDMs by RNAseq. As Tirap^+/-^ and Tirap^-/-^ BMDMs displayed the same resistance phenotype, we performed an analysis to discriminate genes that are commonly modulated in these two backgrounds compared to WT cells during infection (**Fig 3A**). To fine-tune our analysis, we excluded all genes already modulated in non-infected cells (light grey dots on **Fig 4B**). Within 3 hpi, the transcriptional signature in Tirap-deficient BMDMs differed markedly from WT BMDMs with 38 downregulated genes (blue dots) and 62 upregulated genes (orange dots) in Tirap-deficient BMDMs (**Fig 4A and 4B**) (**S1 Table** for the detailed list).

Most genes whose expression was inhibited (e.g. *Il6*, *Il1α*, *Il1β*, *Ifnα* or *Nos2*) are known to be involved in the inflammatory response. Interestingly, *Cish* was previously reported by our team and others to play a role in TB pathogenesis [18]. We confirmed by quantitative RT-PCR that *Cish* expression was 4- and 21-times higher in infected WT BMDMs compared to Tirap^+/-^ and Tirap^-/-^ BMDMs during infection, respectively (**Fig 4C**). Data showed that during Mtb infection, *Cish* is overexpressed and Cish is recruited to MCVs. Sequestration of Cish close to Mtb triggers the ubiquitin-mediated proteasomal degradation of the proton v-ATPase, thus inhibiting the acidification of MCVs and allowing replication of bacteria. Inhibition of *Cish* expression in Tirap-deficient cells during infection could interfere with v-ATPase degradation, and this might explain the capacity of Tirap-deficient cells to control bacterial growth by inducing acidification of MCVs.

In our previous study we showed that infection of macrophages with Mtb leads to secretion of Granulocyte-Macrophage Colony-Stimulating Factor (GM-CSF) inducing STAT5-mediated expression of Cish [18]. To determine whether Tirap participates in intracellular Mtb survival by inducing GM-CSF-dependent STAT5 signaling, we compared *Gmcsf* gene expression during infection between WT and Tirap-deficient BMDMs. In agreement with the expression pattern of *Stat5*, we showed that *Gmcsf* expression was higher in WT BMDMs when compared to expression levels in Tirap-deficient cells (**Fig 4C**). This observation strongly suggests that Tirap acts as a modulator of macrophage defense by triggering STAT5 signaling via GM-CSF secretion. This also supports the hypothesis that Mtb uses Tirap to block the maturation of the vacuole by triggering Cish activity in mutant cells.

To further test this hypothesis, we studied the phenotype of Cish-deficient macrophages to examine whether they act like Tirap-deficient macrophages during Mtb infection. Thus, we investigated the intracellular trafficking of Mtb in RAW264.7 macrophages that were transfected with a lentiviral vector encoding Cish-shRNA (**Fig 4D**). The results show that, similarly to Tirap-deficient macrophages, strong inhibition of *Cish* transcription throughout Mtb infection is associated with decreased bacterial replication, increased phagosomal acidification, blocked phagosomal rupture and reduced amounts of LD in macrophages (**Fig 4E**). Together, our results strongly suggest that the phenotype of Tirap-deficient macrophages is related to Cish-dependent signaling.

### Tirap affects secretion of effectors by intracellular Mtb

**Fig 5:**
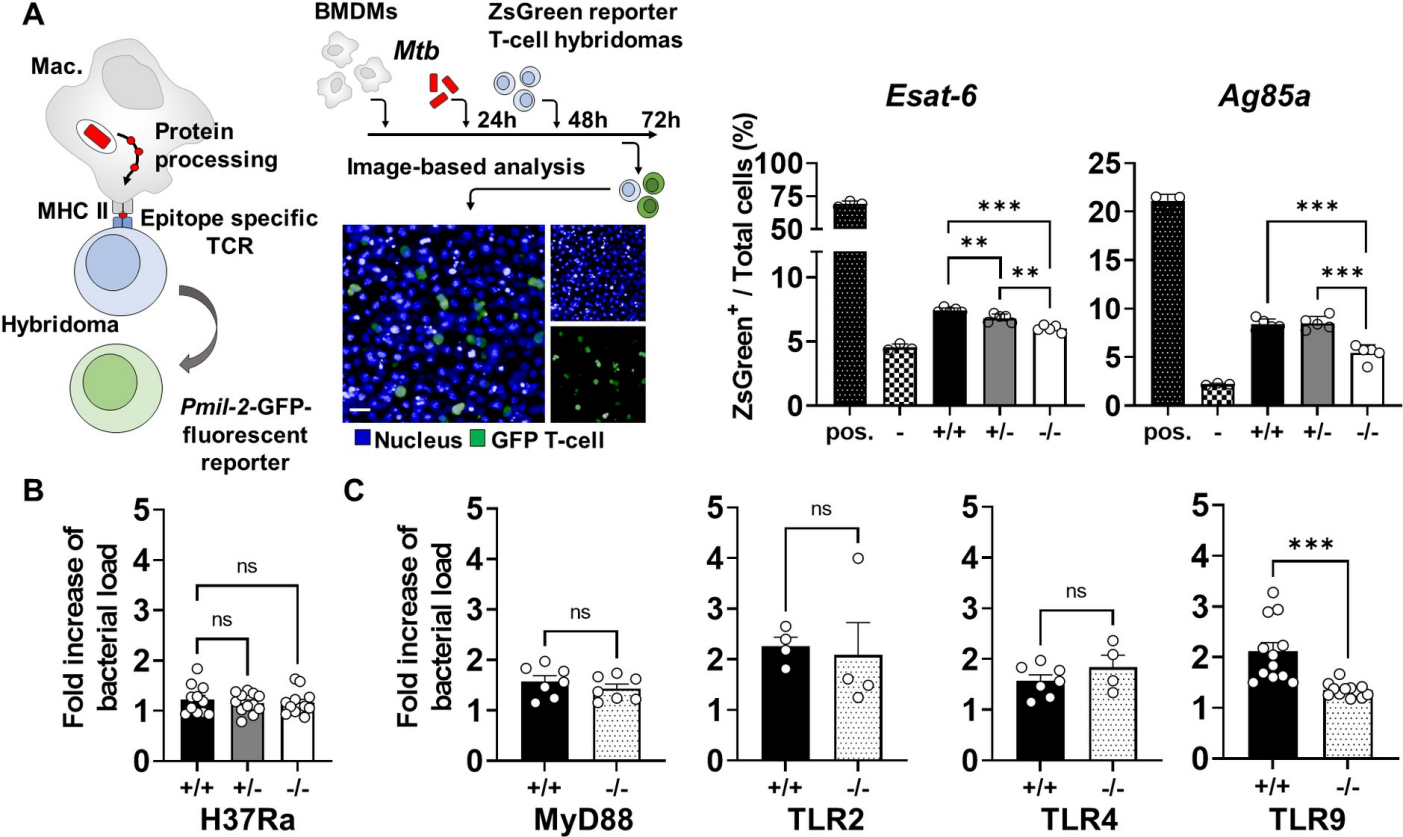
Tirap implication in Mtb effectors secretion. (**A**) BMDMs were seeded into 96-well plates and loaded with homologous (pos) or control (-) peptides or infected with Mtb H37Rv-DsRed. 24 hours post incubation, cells were washed and co-cultured with transduced anti-Ag85 or anti-Esat6 reporter T-cell hybridomas. These were then analyzed by automated confocal microscopy. Shown are representative images of DAPI labelled T-cells (blue) and Antigen specific T-cells (green). Histograms showing mean ± SEM of percentage of ESAT-6 or Ag85 specific T-cells obtained from 5 analysed wells. (**B**) BMDMs from WT, Tirap^+/-^ and Tirap^-/-^ mice were infected with an attenuated strain of Mtb (H37Ra-GFP) (MOI of 2) and analyzed by automated confocal microscopy. The histogram shows the replication fold increase from 3 hpi to 4 dpi. Shown are mean ± SEM obtained from at least 12 analyzed wells per condition. (**C**) WT (+/+), knocked-out (-/-) for Myd88, TLR2, TLR4 and TLR9 BMDMs were infected with H37Rv-GFP (MOI of 2) and analyzed by automated confocal microscopy. The histogram shows the replication fold increase from 3 hpi to 4 dpi. Shown are mean ± SEM obtained from at least 4 analyzed wells per condition of one representative out of two independent experiments. *** P value < 0.001 as determined by one-way ANOVA test.

Our results show that there is a clear evidence that Tirap regulates phagosomal rupture and LD formation. As these events are associated with the secretion of bacterial effectors, we examined the impact of the Tirap genotype on the secretion of ESAT-6 and Antigen 85 (Ag85a) by Mtb within macrophages. We used a previously described multiplexed quantitation tool, which is based on the recognition of effectors of MHC class II epitopes by highly discriminative T cell receptors of a panel of T cell hybridomas [34]. The latter were engineered to express a fluorescent reporter driven by the IL-2 promoter (**Fig 5A**). The results suggested that the secretion of ESAT-6 was slightly decreased in Tirap+/- and Tirap-/- macrophages compared to WT cells (**Fig 5A**). Less Ag85a was also detected in Tirap-/- cells. Together with the lack of replication difference in deficient macrophages infected with Mtb H37Ra (**Fig 5B**), an attenuated strain described to secrete fewer effectors [35], our results suggest that the Tirap-associated phenotype in macrophages could be associated with the expression and secretion of virulence effectors by Mtb. This TB antigen specific hybridoma-based detection assay has the advantage to be a correlate of effectors exported into the cytosol, but it is an indirect detection tool dependent on MHCII antigen processing. In our model, we cannot exclude that Tirap deficiency does not play a role in macrophage antigen presentation. This question needs further in-depth examination, which is beyond the scope of the current study.

### Tirap-associated phenotype is independent of Myd88

We finally considered the impact of Tirap being recruited to the MCV on its implication in signaling pathways of intracellularly expressed TLRs. We investigated the impact of TLR2, TLR4, TLR9 and MyD88 deficiency on Mtb replication (**Fig 5C**). Knock-out BMDMs were infected with Mtb H37Rv-GFP and bacterial growth was quantified at 4 dpi. No replication differences were observed in cells deficient for TLR2, TLR4 and Myd88 compared to WT macrophages. However, we found that TLR9 deficiency restricts Mtb excessive growth and replication at a rate comparable to Tirap deficiency. TLR9 is known to localize to endosomes as well as to phagolysosomes, where it could be triggered by mycobacterial DNA after pathogen uptake. This led us to consider TLR9 as an important candidate pattern-recognition receptor that might account for the Tirap-dependence of host resistance to Mtb. This strongly suggests that the impact of Tirap on Mtb replication could be linked to TLR9 activation in a MyD88-independent signaling pathway, but further investigations are clearly needed. As a matter of fact, Myd88-independent TLR9 signalling was already reported in other studies [36,37].

## Discussion

TLRs are pattern recognition receptors, which sense invading pathogens by recognizing pathogen associated molecular patterns (PAMPs). TLRs recognize several PAMPS of Mtb, such as lipoprotein, lipomannan, lipoarabinomannan and the heat shock protein 65 [38]. PAMP recognition initiates signalling pathways through adaptor proteins (MyD88/Tirap) leading to activation of inflammation. Human studies have indicated that genetic variations in TLR signaling pathway genes regulate the cellular immune response and may influence susceptibility to TB in different populations [39]. Indeed, several studies have focused on the involvement of Tirap polymorphism during Mtb infection leading to conflicting conclusions [11].

In this work, we wanted to study the implication of a deficiency of the TIR domain of Tirap in the control of Mtb infection. To this end, we cross-bred WT and Tirap^-/-^ C57BL/6 mice to generate heterozygous individuals in order to compare the ability of these three mouse genotypes to control intranasal infection with Mtb H37Rv. As previously observed, fully deficient mice control the infection at similar rates as observed in WT mice [40]. However, our results show that heterozygous Tirap-deficient mice are more resistant to infection. This related result illustrates the concept of heterozygote advantage, which corresponds to a more fit heterozygote phenotype compared to homozygotes. This concept suggests that natural selection will maintain polymorphism in the population [41]. This hypothesis could be consistent with human studies showing that heterozygosity for SNP S180L, a loss-of-function associated mutation [10], is also associated with protection against TB [14,22].

In these studies, the authors showed that heterozygous subjects display intermediate inflammatory levels, which is in line with our results.

**Fig 6:**
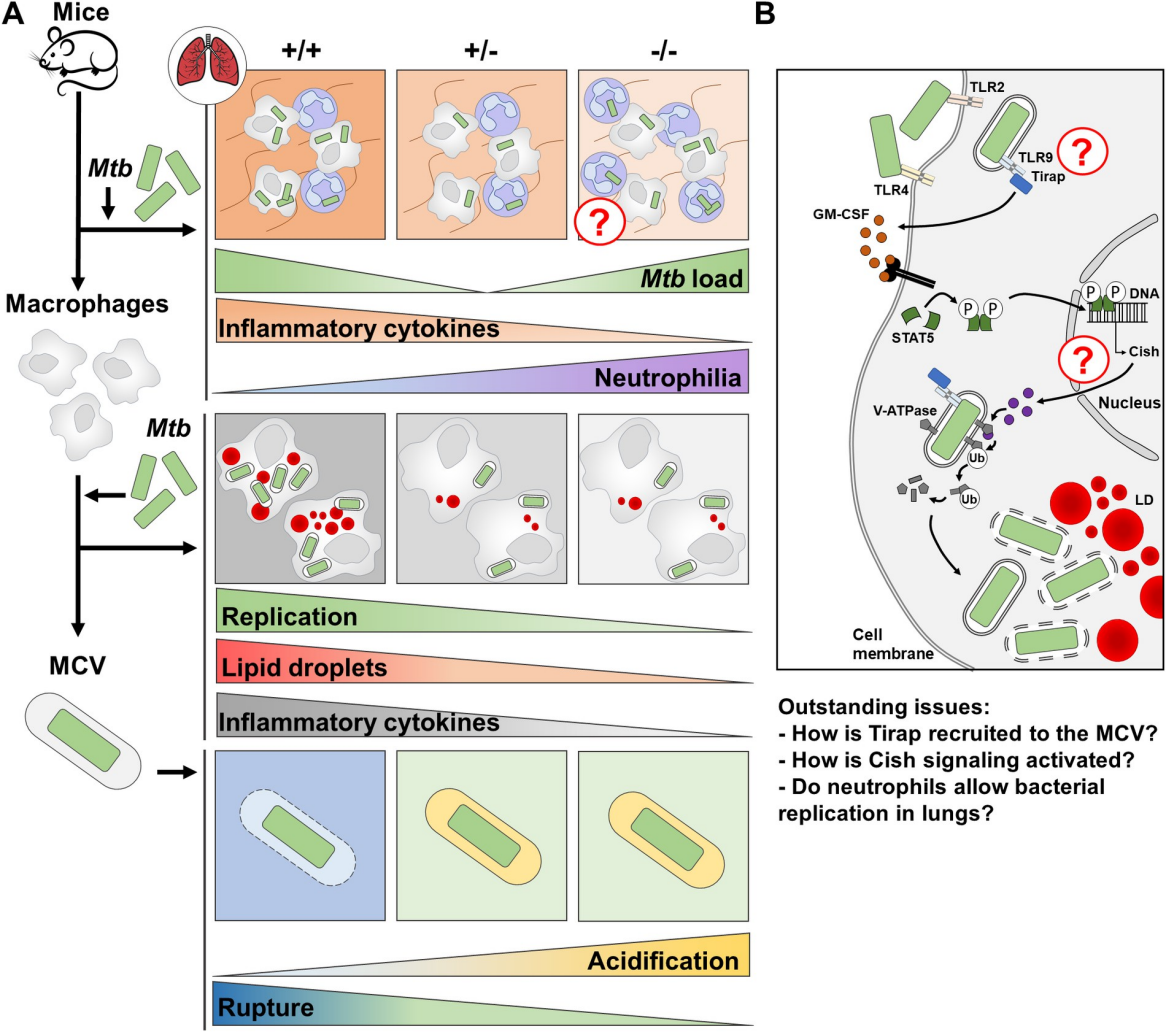
Summary. **(A)** The outcome of Mtb infection is dependent on Tirap expression and depends on whether the mouse has homozygous (susceptibility) or heterozygous (resistance) Tirap KO phenotype. Although homozygous mice show the same susceptibility phenotype, the lungs of WT mice are characterized by a greater inflammatory state than the lungs of completely deficient mice. The latter, in turn, present a neutrophil-rich environment that may be permissive to Mtb replication. At the macrophage level, both homozygous and heterozygous deficiency in Tirap expression restrict intracellular Mtb growth. Efficient killing of the pathogen is promoted by inhibition formation of LD, prevention of production of inappropriate amounts of inflammatory cytokines and induction of a proper MCV maturation. (**B**) Infection of macrophages by Mtb, which leads to the recruitment of Tirap to Mtb-containing vacuoles under WT conditions, limits the acidification of MCVs, induces their rupture and allows Mtb access to the cytosol, where the generation of lipid droplets is induced and fuels the growth of pathogens in host cells. Although our model clearly shows the involvement of the Cish-STAT5 pathway in targeting MCV maturation, the molecular link with the involvement of Tirap remains to be explored.

In fully Tirap-deficient infected mice, we observed a significant increase in the number of recruited pulmonary neutrophils (**Fig 6A** for summary). This strong infiltration may be related to a compensatory and inappropriate response of the host. Sustained neutrophil influx is often associated with susceptibility to Mtb infection [42] especially in genetically susceptible individuals [43,44]. For example, Nair et al. showed that neutrophil neutralization protects against Mtb exacerbation in ACOD-1 deficient mice [45]. We also observed that heterozygous mice displayed a higher level of adaptive immune cells in lungs 28 days post-infection which could explain its phenotype. *In vivo*, these hypotheses need to be further investigated.

In order to decipher the molecular mechanisms involved in this phenotype, we then wanted to specify how Mtb is able to replicate in macrophages, one of the main reservoirs of Mtb replication in the lungs [18]. Using high-content microscopy and image-based analysis, we observed that Tirap deficiency did not affect bacterial uptake but reduced severely Mtb replication levels in heterozygous and full KO macrophages. These observations showed that resistance of heterozygous mice could be correlated with the macrophage phenotype, whereas the resistance of fully deficient macrophages was not reflected in mice. This latter point suggests that despite the difficulty of replication in macrophages, the neutrophil-rich environment and the difficulty in activating inflammatory responses could ultimately allow Mtb replication in the lungs.

Our findings also demonstrate that in wild type macrophages, Mtb infection induces overexpression of Tirap and its recruitment to the MCV. Interestingly, we followed the replication of Mtb in mutant macrophages for TLRs involved in Mtb detection, and we observed that only TLR9 deficiency impairs mycobacterial replication. Together with a human study showing that TLR9 polymorphism is associated with resistance against TB [46], we can expect that Mtb engaged a TLR9/Tirap signalling axis to enable its replication.

Clearly, this hypothesis requires further investigation. Mtb uses efficient strategies to escape eradication by macrophages, such as the recently confirmed escape from phagosomal vacuoles. It was demonstrated that restriction of phagosomal acidification is essential for mycobacterial phagosomal rupture and cytosolic contact [19]. Moreover, LD formation occurs during massive bacterial replication of virulent Mtb. Mtb is thought to trigger LD formation as a strategy to create a host lipid depot to be used as a carbon source reservoir to enable intracellular replication [47]. In our study, we showed that phagosomal acidification is higher in Tirap-deficient cells compared to WT cells. This phenomenon was associated with the absence of phagosomal rupture and LD formation (**Fig 6A** for summary). Interestingly, inhibition of intracellular acidification using ConA restores a WT phenotype in deficient cells exhibiting strong phagosomal rupture and LD formation. The ESX-1 type VII secretion system of Mtb governs numerous aspects of the host-pathogen interaction thought to be implicated in phagosomal maturation. The host cell factors governing these events are yet unexplored and require further investigation, which is of importance to determine whether Tirap may be one of them.

In order to identify the mechanism by which Mtb uses Tirap signalling to promote its replication, we performed a RNAseq analysis to compare gene expression between infected Tirap-deficient and WT macrophages. Among the hundred genes identified, the *Cish* gene was subject to an interesting downregulation in mutant macrophages. A previous study from our group showed that the v-ATPase is targeted for ubiquitination and proteasomal degradation during Mtb infection due to the expression of Cish [18]. V-ATPase degradation prevents MCV acidification and promotes Mtb replication. More precisely, Mtb infection leads to granulocyte-macrophage colony-stimulating factor (GM-CSF) secretion, inducing STAT5-mediated expression of Cish. Consistently, it was shown that inhibition of Cish expression led to reduced replication of Mtb in macrophages. Therefore, we monitored GM-CSF and STAT5 expression in Tirap-deficient cells. Interestingly, reduced rexpression of Cish was associated with a lower expression level of GM-CSF and STAT5 in both heterozygous and homozygous Tirap KO macrophages (**Fig 6B** for summary). To decipher whether reduced Cish expression could be associated with the phenotype observed in Tirap-deficient mice, Cish was knocked down in macrophages using a shRNA-encoding lentiviral vector. Interestingly, Cish inhibition produced results similar to those observed in Tirap-deficient cells regarding bacterial replication, phagosomal acidification, cytosolic access and LD formation. From those experiments, we conclude that Tirap and Cish deficiency induce similar phenotypes upon Mtb infection. More investigations are needed to determine if the two signalling are directly related.

Overall, our results show that only Tirap heterozygous mice are more resistant to Mtb infection, which correlates with a substantial number of studies showing that heterozygous polymorphism associated with a Tirap loss-of-function confers resistance to Mtb infection in humans.

However, a previous study in mice investigated the role of the Tirap S200L mutation (which correspond to the common S180L human variant) during Mtb infection [48]. The authors showed that this mutation impairs *in vitro* and *in vivo* responses to the infection, which is associated with an increased bacterial survival. This mutation attenuates IFNγ signaling that affect phagosomal maturation and autophagy. This research pint-points that the single nucleotide S200L polymorphism induces an opposite phenotype than the full lack of the *Tirap* gene observed in our work. Besides the fact that experimental procedures (e.g. higher MOI, IFN-γ macrophage activation, protocol of infection) are quite different between the two studies, it is of interest to look at the relevance of the two models. In our model we mainly wanted to investigate the impact of a total or partial lack of Tirap to decipher its fundamental role in the control of tuberculosis, which is relevant to understand the biology of the protein. The identification of host genetic markers remains useful to predict TB development and understand the immunopathogenesis of the disease. In this context, our work reveals the potential of Tirap inhibition as an attractive target for the development of new therapeutic strategies. The second model is also of interest as it mimics a common human variant of Tirap described to play a role on the control of the infection. It is well known that Tirap is involved in numerous intracellular processes that could be modulated by a single point mutation. Together, these two studies need to be considered as complementary, thus strengthening the importance that further investigations are needed to decipher the role of Tirap downstream signalling during Mtb infection.

## Material and methods

### Ethics statement

All mice were maintained, and breeding was performed in the animal facility of the Pasteur Institute of Lille, France, together with heterozygous mice for TIRAP gene (Tirap^+/-^) generated there (agreement B59-350009). All experimental procedures were approved by the institutional ethical committee “Comité d’Ethique en Experimentation Animale (CEEA) 75, Nord Pas-de-Calais” and the “Education, Research and Innovation Ministry” (APAFIS#1 327 0232–2017061411305485 v6, approved on 14/09/2018). All experiments were performed in accordance with relevant guidelines and regulations.

### Reagents

Cell nuclei were fluorescently labelled using DAPI (Sigma Aldrich) or Hoechst 33342 (ThermoFisher). Lysotracker Green DND-26, CCF4-AM and LipidTox Deep Red were obtained from ThermoFisher.

### Mice

C57BL/6NJ wild type and Tirap^-/-^ mice were purchased from The Jackson Laboratory (Bar Harbor, ME, USA). Mice genotyping was performed using the KAPA Taq EXtra HotStart ReadyMix PCR Kit according to the manufacturer instructions (Biosystems). The following primer pairs were used to amplify a 900 bp DNA fragment of the wild type (a+b) and the mutant genes (b+c). Primer a CATCCTGTGTGGCTGTCTGTGAACCAT, Primer b TGGCCAATGTGTGAGCAAGTTCTGTGC, Primer c ATCGCCTTCTATCGCCTTCTTGACGAG.

### Murine bone marrow-derived macrophages (BMDMs)

Murine bone-marrow progenitors were obtained by sampling tibias and femur bones from 8 to 12-week-old C57BL/6NJ wild type, Tirap^+/-^ and Tirap^-/-^ mice. BMDMs were obtained by seeding 10^7^ bone marrow cells in 75 cm^2^ flasks in RPMI 1640 Glutamax medium (Gibco) supplemented with 10% heat-inactivated Fetal Bovine Serum (FBS, Gibco; RPMI-FBS) and 10% L929 cell supernatant containing Macrophage Colony-Stimulating Factor (M-CSF). Fresh medium was added every 3–4 days. After 7 days incubation at 37°C in an atmosphere containing 5% CO2, the BMDMs monolayer was rinsed with D-PBS and cells were harvested with Versene (Gibco). BMDM were resuspended into culture medium to be used for subsequent assays.

### Bacteria

Recombinant strains of Mtb H37Rv expressing an enhanced green fluorescent protein (GFP) or a red fluorescent protein DsRed [49] were cultured in Middlebrook 7H9 medium (Difco) supplemented with 10% oleic acid-albumin-dextrose-catalase (OADC, Difco), 0.2% glycerol (Euromedex), 0.05% Tween 80 (Sigma-Aldrich) and 50 μg/ml hygromycin (ThermoFisher Scientific) or 25 μg/ml kanamycin (Sigma-Aldrich) for H37Rv-GFP or H37Rv-DsRed, respectively. Cultures were maintained for 14 days until the exponential phase was reached. Before cell infection, bacilli were washed with Dulbecco’s Phosphate Buffered Saline (DPBS, free from MgCl2 and CaCl2, Gibco), resuspended in 10 mL RPMI-FBS and centrifuged at 1000 RPM for 2 min at room temperature to remove bacterial aggregates. Bacterial titer of the suspension was determined by measuring the optical density (OD600 nm) and GFP or DsRed fluorescence on a Victor Multilabel Counter (Perkin Elmer). The bacterial suspension was diluted at the required titre in RPMI 1640 supplemented with 10% FBS prior to infection. For *in vivo* studies, the non-fluorescent Mtb H37Rv strain were grown in Middlebrook 7H9 medium, as described previously [50,51].

### Infection of mice and determination of bacterial burden

8-12-week-old mice were inoculated with H37Rv via the intranasal route (i.n.) (10^5^ CFU/20 μL) as previously described [52]. 28 days post infection, mice were euthanized, and lungs were harvested for bacterial burden evaluation by colony forming units (CFU) enumeration. Lungs were homogenized for 20 min in a tube containing 2.5 mm diameter glass beads and 1 ml of PBS using the MM 400 mixer mill (Retsch GmbH, Haan, Germany). Ten-fold serial dilutions (from 10^−2^ to 10^−9^) of each sample were plated onto 7H11 medium agar plate (Difco) supplemented with 10% oleic acid-albumin-dextrose-catalase (OADC, Difco). After a 2-week growth period at 37°C, CFUs were determined at the appropriate dilution allowing optimal colonies enumeration.

### Lung histopathology

At the determined time-point, mice were euthanized, lungs were harvested perfused and soaked in 4% formaldehyde (10% formalin solution, neutral buffered, HT501128, Sigma-Aldrich) for 24 hours at 4°C and then dehydrated in a series of ethanol solutions to visualize their internal structures. Specimens were then embedded and cut in sections down to 5 μm thickness. Slices were colored using toluidine blue (0.1%) for 4 min after being dewaxed and rehydrated. Samples were examined using an optical microscope (Zeiss Axio lab A1) and a stereomicroscope (Zeiss Stemi 305) and a camera and the Zen 2011 module (Zeiss) for image analyses.

### Flow cytometry

A described previously [53], lungs were harvested, cut into small pieces and incubated for 1 hour at 37°C with a mix of DNAse I (100 μg/ml, Sigma-Aldrich) and collagenase (400 U/ml 1.6 mg/ml, Roche). Lung cells were washed and filtered through a 100 μM filter before being incubated with saturating doses of purified 2.4G2 (anti-mouse Fc receptor, ATCC) in 200 μL PBS 0.2% BSA 0.02% NaN3 (FACS buffer) for 20 minutes at 4°C to prevent antibody binding on the Fc receptor. Various fluorescent mAb combinations in FACS buffer were used to determine cell populations (Table 1). Acquisitions were done on FACScanto II cytofluorometer (Becton Dickinson) with the following mAbs from BD Biosciences: Fluorescein (FITC)-coupled HL3 (anti-CD11c), FITC-coupled 145-2C11 (anti-CD3), APC-coupled RB6-8C5 (anti-GR1), phycoerythrine (PE)-coupled RM4-5 (anti-CD4), PE-coupled E50-2440 (anti-SIGLEC-F), APC-coupled BM8 (anti-F4/80). APC-eF780-coupled M1/70 (antiCD11b) were purchased from eBiosciences and fixable viability dye Aqua (ThermoFisher) was used to gate viable cells. Gating strategies are summarized in **S3 Fig**.

**Table 1:** Phenotypic determination of pulmonary immune cells.

Cell type	Phenotype
Neutrophils	CD11b^+^ Ly6G^+^
Dendritic cells	CD11b^+^ CD11c^+^ F4/80^-^
Alveolar macrophages	F4/80^+^ SiglecF^+^ CD11c^+^
Interstitial macrophages	F4/80^+^ SiglecF^-^ CD11c^int^
CD4 T cells	CD3^+^ CD4^+^
CD8 T cells	CD3^+^ CD8^+^
B cells	CD3^-^ B220^+^ MHCII^+^

### Infection for quantification of intracellular mycobacterial replication, phagosomal acidification, phagosomal rupture and Lipid Droplets (LD) formation

2x10^4^ BMDM were seeded per well in 384-well plates. Cells were infected for 3 h with H37Rv-GFP at a MOI of 2. Cells were extensively washed with RPMI-FBS in order to remove extracellular Mtb and incubated at 37°C with 5% CO_2_.

For intracellular mycobacterial replication assay, 10% formalin solution (Sigma-Aldrich) containing 10 μg/mL Hoechst 33342 (Life-Technologies) was added to each well at 3 h and 96 hpi. Plates were incubated for 30 min, allowing nuclei staining and cell fixation. Cells were stored in DPBS until image acquisition.

To quantify phagosomal acidification 3 hpi, cells were incubated with 1mM LysoTracker Green DND-26 for 1.5h at 37°C with 5% CO_2_. Cells were then fixed with a solution containing 10% formalin solution and 10 μg/mL Hoechst 33342 [25].

To inhibit intracellular acidification, cells were incubated with 100 nM of Concanamycin A (ConA) (Sigma-Aldrich, C9705) 2 h before infection until the end of the assay.

For phagosomal rupture, 24 hpi, cells were stained with 8 μM CCF4-AM in EM buffer (120 mM NaCl2, 7 mM KCl, 1.8 mM CaCl2, 0.8 mM MgCl2, 5 mM glucose, 2.5 μM probenecid, and 25 mM Hepes, pH 7.3) for 1 h at room temperature in the dark. Cells were then washed three times using EM buffer before imaging.

For LD formation assay, cells were washed and fixed at 96 hpi, as previously described [25]. Cells were washed twice with DBPS and intracellular LD were stained with 25 μL per well of 2000-fold diluted HCS LipidTOX deep Red neutral lipid probe (Invitrogen) in DPBS for 30 min at room temperature.

### Image acquisition

Images were acquired using an automated fluorescent confocal microscope (In Cell analyzer 6000, GE) equipped with a 20X (NA 0.70) air lens or 60X (NA 1.2) water lens for Tirap localization, intracellular mycobacterial replication, phagosomal acidification, phagosomal rupture, LD formation and quantitation of intracellular Mtb secreted effectors assays. The confocal microscope was equipped with 405, 488, 561 and 642 nm excitation lasers. The emitted fluorescence was captured using a camera associated with a set of filters covering a detection wavelength ranging from 450 to 690 nm. Hoechst 33342-stained nuclei and CCF4-stained cells were detected using the 405 nm laser with a 450/50-nm emission filter. Green signals corresponding to LysoTracker Green DND-26, CCF4-AM, ZsGreen^+^ Tcells, and H37Rv-GFP were recorded using 488 nm laser with 540/75-nm emission filters. Red signals corresponding to H37Rv-DsRed was recorded using 561 nm laser with 600/40-nm emission filters. LipidTOX signal was detected using 630-nm excitation and 690-nm emission wavelengths.

### Image analysis

Images from the automated confocal microscope were analyzed using multi-parameter scripts developed using Columbus system (version 2.3.1; PerkinElmer). Segmentation algorithms were applied to input images to detect nuclei and the signal of Mtb H37Rv to determine infection and replication rates. Briefly, the host cell segmentation was performed using two different Hoechst signal intensities—a strong intensity corresponding to the nucleus and a weak intensity in cytoplasm—with the algorithm “Find Nuclei” and “Find Cytoplasm”, as described previously [25]. GFP or DsRed signal intensities in a cell were used for the intracellular bacterial segmentation with the algorithm “Find Spots”. The identified intracellular bacteria were quantified as intracellular Mtb area with number of pixels. Subsequently, the population of infected cells was determined, and the increase of intracellular Mtb area, corresponding to intracellular mycobacterial replication, was calculated. For quantification of phagosomal acidification with Lysotracker Green DND-26, green signal intensity in a cell was used for the intracellular acidic compartment segmentation with the algorithm “Find Spots”.

### Assessment of intracellular mycobacterial load by CFU plating

BMDMs were grown at 1x10^5^ cells/well in 24-well plates in RPMI supplemented with 10% FBS and 10% M-CSF. Macrophages were infected with Mtb H37Rv (MOI of 2). Extracellular bacteria were washed off at 3 hr post-infection. At indicated time points post-infection, cells were lysed and five-fold serial dilutions of each sample were plated onto 7H11 medium agar plates (Difco) supplemented with 10% oleic acid-albumin-dextrose-catalase (OADC, Difco). After a 2-week growth period at 37°C, CFUs were determined at the appropriate dilution allowing optimal enumeration of colonies.

### Recognition of effectors MHC class II epitopes by highly discriminative T cell hybridomas

As previously described [34], BMDMs were seeded into 96-well plates at 1 × 10^5^ cells/well in 100 μL of RPMI containing 10% FBS and 10% M-CSF. After overnight incubation, cells were loaded with 1 μg/mL of homologous or control peptides or infected with Mtb H37Rv-DsRed (MOI of 2). 24 hpi, cells were washed and co-cultured with 5 × 10^4^ transduced anti-Ag85 or anti-Esat6 T cell hybridomas. After 24 h incubation, the non-adherent T cells were transferred into new 96-well plates. Cell nuclei were stained with 10 μg/mL of Hoechst 33342 (Sigma-Aldrich) for 30 min at 37°C. Image acquisitions were performed on an automated fluorescence confocal microscope (InCell 6500) using a 20x lens. Hoechst-labelled T cell nuclei were detected using a 405-nm excitation laser coupled with a 450/50 detection filter, and ZsGreen^+^ antigen-activated T cells were detected using a 488-nm laser coupled with a 540/75 detection filter.

### Construction of a lentivirus expressing TIRAP-mCherry

The coding sequence of TIRAP-GFP was amplified by PCR using primers 5’- TTTGAGATCTACCATGGCATCATCGACCTCC-3’ and 5’- TTTGCTCGAGTTACTTGTACAGCTCGTCCATG-3’ and the plasmid Tirap-GFP PMSCV2.2-KCD2, kindly provided by Jonathan Kagan, as a template. The PCR product was restricted with BglII and XhoI and inserted in the plasmid pRRL.sin.cPPT.SFFV/IRES-puro.WPRE plasmid [54], restricted with BamHI and XhoI. Then, the GFP coding sequence was excised from the resulting construct using BamHI and BsrGI, and exchanged for that of mCherry, which had been amplified by PCR using primers 5’- TTTGGGATCCACCGGTCGCCACCATGGTGAGCAAGGGCGA-3’ and 5’- TTTGGAGCTCTTACTTGTACAGCTCGTCCATGC -3’. A lentiviral vector stock was obtained by cotransfection of HEK-293T cells with the resulting plasmid (pRRL.sin.cPPT.SFFV/TIRAP-mCherry.IRES-puro.WPRE) and plasmids expressing HIV Gag-Pol and VSV-G at a ratio of 5:4:1. The culture medium was collected after 3 days at 33°C, filtered and used to transduce RAW264.7 cells for 48 hours to study Tirap intracellular distribution in Mtb infected cells. After 2 hours of infection with Mtb H37Rv-GFP, cells were imaged using a confocal microscope (Zeiss LSM880) equipped with a 40x objective and Zen imaging software (Zeiss, Germany). The images were analysed using ImageJ software.

### Lentiviral transduction

RAW264.7 cells were transduced with lentiviral vector expressing Cish targeting shRNA (NM_009895, openbiosystems) for 48 hours. Transduced cells were then selected with 10 μg/mL of puromycin. The inhibition of Cish transcription was validated by quantitative RT-PCR.

### RNA extraction

BMDMs were grown in 6-well plates and RNA was extracted using QIAzol lysis reagent and miRNeasy Mini Kit according to the manufacturer instructions (Qiagen). RNA concentration was determined using the GE SimpliNano device (GE Healthcare, UK). Remaining DNA in samples was digested using the amplification grade DNase I kit (Sigma-Aldrich, USA) for 6 min at RT. The reaction was stopped by heat inactivation for 10 min at 70°C.

Total RNA from lung tissues were extracted with the NucleoSpin RNA kit (Macherey-Nagel, Hoerdt, Germany).

### Quantitative RT-PCR

For BMDMs RNA extracts, cDNA synthesis was achieved by reverse transcription using the Superscript IV Vilo Mastermix kit (ThermoFisher, USA) following the manufacturer’s instructions. RNA from lung extract was reverse-transcribed with the High-Capacity cDNA Archive Kit (Life Technologies, USA). qPCR was performed using the applied biosystems SYBR Select Master Mix (Thermofischer) with 20 ng cDNA per sample and the appropriate primer pairs (Table 2). Gapdh was used as the reference housekeeping gene for normalization. qPCR reactions were measured by the QuantStudio 12K Flex system (Applied Biosystems, USA) using the following cycles: 2 min 50°C, 10 min 95°C followed by 40 cycles of 15 s 95°C, 30 s 60°C and 30 s 72°C. The target mRNA fold change was calculated based on the 2-ΔΔCt formula, where the Gapdh gene was used as the reference gene, and RNA from non-infected wild type BMDMs was used as the standard condition.

**Table 2:** Forward and reverse primers sequences.

Genes	Forward	Reverse
*Arg1*	ATTGTGAAGAACCCACGGTCTG	ACTGTGGTCTCCACCCAGCA
*Cish*	CTAGACCCTGAGGGGGATCT	GGGTGCTGTCTCGAACTAGG
*Gapdh*	GCAAAGTGGAGATTGTTGCCA	GCCTTGACTGTGCCGTTGA
*Gmcsf*	TGCCTGTCACGTTGAATGAAGA	CCCGTAGACCCTGCTCGAATA
*Ifng*	CAACAGCAAGGCGAAAAAG	GTGGACCACTCGGATGAGCT
*Il12p40*	GACCCTGCCCATTGAACTGGC	CAACGTTGCATCCTAGGATCG
*Il1b*	TCGTGCTGTCGGACCCATA	GTCGTTGCTTGGTTCTCCTTGT
*Il6*	CAACCACGGCCTTCCCTACT	CCACGATTTCCCAGAGAACATG
*Inos*	CAGCTGGGCTGTACAAACCTT	CATTCGAAGTGAAGCGTTTCG
*Tnfa*	CATCTTCTCAAAATTCGAGTGACAA	TGGGAGTAGACAAGGTACAACCC
*Tirap*	CCTCCACTCCGTCCAAGAAG	TGAACCATCATAGAGGTGGCTTT

### RNA sequencing

RNA quality was analyzed by the measurement of the RNA integrity number (RIN) with a bioanalyzer RNA 6000 Nano assay prior to sequencing. mRNA library preparation was realized following manufacturer’s recommendations (Ultra 2 mRNA kit from NEB). Final samples pooled library prep were sequenced on Novaseq6000 ILLUMINA with S1-200 cartridge (2x1600Millions of 100 bases reads) in one run, corresponding to 2x30Millions of reads per sample after demultiplexing. Quality of raw data was evaluated with FastQC. Poor quality sequences and adapters were trimmed or removed with the fastp tool, using default parameters, to retain only good quality paired reads. Illumina DRAGEN bio-IT Plateform (v3.8.4) was used for mapping on mm10 reference genome and for quantification using gencode vM25 annotation gtf file. Library orientation, library composition and coverage along transcripts were checked with Picard tools. Subsequent analyses were conducted with R software. Differential expression analysis was performed with the DESeq2 (v1.26.0) bioconductor package. Multiple hypothesis adjusted p-values were calculated with the Benjamini-Hochberg procedure to control FDR with a threshold of significance at 0.05. The cut-off for absolute log2-ratio was set at 2.

### ELISA assay

Cytokine production was measured from lung extracts (28 dpi) or BMDMs supernatants (4 dpi) accordingly following protocol recommendations of the manufacturer for IL-1β, IL-12p40, IFNγ (Invitrogen—Waltham, MA) and TNFα (R&D Systems—Minneapolis, MN).

### Statistics

All analyses and histograms were performed using GraphPad Prism 9 software. Significance of obtained results was tested using Ordinary one-way ANOVA. In **S2 Fig**, differences in the mean between two groups were analyzed using Student’s t-test. Indicated symbols of *, **, *** and **** denote p < 0.05, p < 0.01, p < 0.001 and p < 0.0001 respectively.

## Supporting information

S1 Fig (A) Workflow of mycobacterial *in vivo* and *in vitro* infection experiments indicating different read-outs. (B) Comparison of the number of resident immune cells in the lungs of 4 naive mice for each mouse genotype. Cell numbers were normalized to the total cell number analyzed in each sample and were extrapolated to the whole lung. Shown are mean ± SEM of cells obtained in each group. (C) Comparison of BMDMs proliferation from the three mouse genotypes. * P value < 0.05, ** P value < 0.01, as determined by one-way ANOVA test.
(TIF)

S2 Fig (A) Histograms showing fold increase in different cytokine expression in lungs of infected +/+, +/- and -/- mice. Shown are mean ± SEM of 5 infected mice per condition. (B) Histograms showing mean ± SEM of fold increase in different cytokine expression in BMDMs from +/+, +/- and -/- mice. Results are shown from one representative out of two independent experiments.
(TIF)

S3 Fig Gating strategies applied in flow cytometry to analyze the different populations in lungs of mice. (TIF)

S1 Table Detailed list of the genes modulated in infected deficient cells (+/- and -/-) compared to WT non-infected macrophages. (XLSX)

## References

[1] Barreiro LB, Ben-Ali M, Quach H, Laval G, Patin E, Pickrell JK, et al. Evolutionary dynamics of human Toll-like receptors and their different contributions to host defense. PLoS Genet. 2009;5(7):e1000562. doi: 10.1371/journal.pgen.1000562 19609346 PMC2702086

[2] Noreen M, Arshad M. Association of TLR1, TLR2, TLR4, TLR6, and TIRAP polymorphisms with disease susceptibility. Immunol Res. 2015;62(2):234–52. doi: 10.1007/s12026-015-8640-6 25784622

[3] D’Onofrio V, Monnier AA, Kremer C, Stappers MHT, Netea MG, Gyssens IC. Lesion size is associated with genetic polymorphisms in TLR1, TLR6, and TIRAP genes in patients with major abscesses and diabetic foot infections. Eur J Clin Microbiol Infect Dis. 2020;39(2):353–60. doi: 10.1007/s10096-019-03732-7 31786695 PMC7010613

[4] Collin M, Dickinson R, Bigley V. Haematopoietic and immune defects associated with GATA2 mutation. Br J Haematol. 2015;169(2):173–87. doi: 10.1111/bjh.13317 25707267 PMC4409096

[5] Rajpoot S, Wary KK, Ibbott R, Liu D, Saqib U, Thurston TLM, et al. TIRAP in the Mechanism of Inflammation. Front Immunol. 2021;12:697588. doi: 10.3389/fimmu.2021.697588 34305934 PMC8297548

[6] Zyzak J, Mitkiewicz M, Leszczyńska E, Reniewicz P, Moynagh PN, Siednienko J. HSV-1/TLR9-Mediated IFNβ and TNFα Induction Is Mal-Dependent in Macrophages. J Innate Immun. 2020;12(5):387–98. 31851971 10.1159/000504542 PMC7506264

[7] Takeda K, Akira S. Microbial recognition by Toll-like receptors. J Dermatol Sci. 2004;34(2):73–82. doi: 10.1016/j.jdermsci.2003.10.002 15033189

[8] Jeyaseelan S, Manzer R, Young SK, Yamamoto M, Akira S, Mason RJ, et al. Toll-IL-1 receptor domain-containing adaptor protein is critical for early lung immune responses against Escherichia coli lipopolysaccharide and viable Escherichia coli. J Immunol. 2005;175(11):7484–95. doi: 10.4049/jimmunol.175.11.7484 16301656

[9] Jeyaseelan S, Young SK, Yamamoto M, Arndt PG, Akira S, Kolls JK, et al. Toll/IL-1R domain-containing adaptor protein (TIRAP) is a critical mediator of antibacterial defense in the lung against Klebsiella pneumoniae but not Pseudomonas aeruginosa. J Immunol. 2006;177(1):538–47. doi: 10.4049/jimmunol.177.1.538 16785551

[10] Khor CC, Chapman SJ, Vannberg FO, Dunne A, Murphy C, Ling EY, et al. A Mal functional variant is associated with protection against invasive pneumococcal disease, bacteremia, malaria and tuberculosis. Nat Genet. 2007;39(4):523–8. doi: 10.1038/ng1976 17322885 PMC2660299

[11] Belhaouane I, Hoffmann E, Chamaillard M, Brodin P, Machelart A. Paradoxical Roles of the MAL/Tirap Adaptor in Pathologies. Front Immunol. 2020;11:569127. doi: 10.3389/fimmu.2020.569127 33072109 PMC7544743

[12] Saranathan R, Sathyamurthi P, Thiruvengadam K, Murugesan S, Shivakumar S, Gomathi NS, et al. MAL adaptor (TIRAP) S180L polymorphism and severity of disease among tuberculosis patients. Infect Genet Evol. 2020;77:104093. doi: 10.1016/j.meegid.2019.104093 31678649

[13] Liu Q, Li W, Li D, Feng Y, Tao C. TIRAP C539T polymorphism contributes to tuberculosis susceptibility: evidence from a meta-analysis. Infect Genet Evol. 2014;27:32–9. doi: 10.1016/j.meegid.2014.06.025 25003251

[14] Castiblanco J, Varela DC, Castaño-Rodríguez N, Rojas-Villarraga A, Hincapié ME, Anaya JM. TIRAP (MAL) S180L polymorphism is a common protective factor against developing tuberculosis and systemic lupus erythematosus. Infect Genet Evol. 2008;8(5):541–4. 18417424 10.1016/j.meegid.2008.03.001

[15] Miao R, Li J, Sun Z, Xu F, Shen H. Meta-analysis on the association of TIRAP S180L variant and tuberculosis susceptibility. Tuberculosis (Edinb). 2011;91(3):268–72. doi: 10.1016/j.tube.2011.01.006 21419702

[16] WHO. Global Tuberculosis Report 2021. 2021.

[17] Kinsella RL, Zhu DX, Harrison GA, Mayer Bridwell AE, Prusa J, Chavez SM, et al. Perspectives and Advances in the Understanding of Tuberculosis. Annu Rev Pathol. 2021;16:377–408. doi: 10.1146/annurev-pathol-042120-032916 33497258

[18] Queval CJ, Song OR, Carralot JP, Saliou JM, Bongiovanni A, Deloison G, et al. Mycobacterium tuberculosis Controls Phagosomal Acidification by Targeting CISH-Mediated Signaling. Cell Rep. 2017;20(13):3188–98. doi: 10.1016/j.celrep.2017.08.101 28954234 PMC5637157

[19] Simeone R, Sayes F, Song O, Gröschel MI, Brodin P, Brosch R, et al. Cytosolic access of Mycobacterium tuberculosis: critical impact of phagosomal acidification control and demonstration of occurrence in vivo. PLoS Pathog. 2015;11(2):e1004650. doi: 10.1371/journal.ppat.1004650 25658322 PMC4450080

[20] Sánchez D, Lefebvre C, Rioux J, García LF, Barrera LF. Evaluation of Toll-like receptor and adaptor molecule polymorphisms for susceptibility to tuberculosis in a Colombian population. Int J Immunogenet. 2012;39(3):216–23. doi: 10.1111/j.1744-313X.2011.01077.x 22221660

[21] Selvaraj P, Harishankar M, Singh B, Jawahar MS, Banurekha VV. Toll-like receptor and TIRAP gene polymorphisms in pulmonary tuberculosis patients of South India. Tuberculosis (Edinb). 2010;90(5):306–10. doi: 10.1016/j.tube.2010.08.001 20797905

[22] Capparelli R, De Chiara F, Di Matteo A, Medaglia C, Iannelli D. The MyD88 rs6853 and TIRAP rs8177374 polymorphic sites are associated with resistance to human pulmonary tuberculosis. Genes Immun. 2013;14(8):504–11. doi: 10.1038/gene.2013.48 24067789

[23] Nejentsev S, Thye T, Szeszko JS, Stevens H, Balabanova Y, Chinbuah AM, et al. Analysis of association of the TIRAP (MAL) S180L variant and tuberculosis in three populations. Nat Genet. 2008;40(3):261–2; author reply 2–3. doi: 10.1038/ng0308-261 18305471

[24] Yamamoto M, Sato S, Hemmi H, Sanjo H, Uematsu S, Kaisho T, et al. Essential role for TIRAP in activation of the signalling cascade shared by TLR2 and TLR4. Nature. 2002;420(6913):324–9. doi: 10.1038/nature01182 12447441

[25] Deboosere N, Belhaouane I, Machelart A, Hoffmann E, Vandeputte A, Brodin P. High-Content Analysis Monitoring Intracellular Trafficking and Replication of Mycobacterium tuberculosis Inside Host Cells. Methods Mol Biol. 2021;2314:649–702. doi: 10.1007/978-1-0716-1460-0_29 34235675

[26] Hoffmann E, Machelart A, Song OR, Brodin P. Proteomics of Mycobacterium Infection: Moving towards a Better Understanding of Pathogen-Driven Immunomodulation. Front Immunol. 2018;9:86. doi: 10.3389/fimmu.2018.00086 29441067 PMC5797607

[27] Barczak AK, Avraham R, Singh S, Luo SS, Zhang WR, Bray MA, et al. Systematic, multiparametric analysis of Mycobacterium tuberculosis intracellular infection offers insight into coordinated virulence. PLoS Pathog. 2017;13(5):e1006363. doi: 10.1371/journal.ppat.1006363 28505176 PMC5444860

[28] Augenstreich J, Arbues A, Simeone R, Haanappel E, Wegener A, Sayes F, et al. ESX-1 and phthiocerol dimycocerosates of Mycobacterium tuberculosis act in concert to cause phagosomal rupture and host cell apoptosis. Cell Microbiol. 2017;19(7). doi: 10.1111/cmi.12726 28095608

[29] Conrad WH, Osman MM, Shanahan JK, Chu F, Takaki KK, Cameron J, et al. Mycobacterial ESX-1 secretion system mediates host cell lysis through bacterium contact-dependent gross membrane disruptions. Proc Natl Acad Sci U S A. 2017;114(6):1371–6. doi: 10.1073/pnas.1620133114 28119503 PMC5307465

[30] Song OR, Deboosere N, Delorme V, Queval CJ, Deloison G, Werkmeister E, et al. Phenotypic assays for Mycobacterium tuberculosis infection. Cytometry A. 2017;91(10):983–94. doi: 10.1002/cyto.a.23129 28544095

[31] Simeone R, Bobard A, Lippmann J, Bitter W, Majlessi L, Brosch R, et al. Phagosomal rupture by Mycobacterium tuberculosis results in toxicity and host cell death. PLoS Pathog. 2012;8(2):e1002507. doi: 10.1371/journal.ppat.1002507 22319448 PMC3271072

[32] den Brok MH, Raaijmakers TK, Collado-Camps E, Adema GJ. Lipid Droplets as Immune Modulators in Myeloid Cells. Trends Immunol. 2018;39(5):380–92. doi: 10.1016/j.it.2018.01.012 29478771

[33] Huss M, Ingenhorst G, König S, Gassel M, Dröse S, Zeeck A, et al. Concanamycin A, the specific inhibitor of V-ATPases, binds to the V(o) subunit c. J Biol Chem. 2002;277(43):40544–8. 12186879 10.1074/jbc.M207345200

[34] Sayes F, Blanc C, Ates LS, Deboosere N, Orgeur M, Le Chevalier F, et al. Multiplexed Quantitation of Intraphagocyte Mycobacterium tuberculosis Secreted Protein Effectors. Cell Rep. 2018;23(4):1072–84. doi: 10.1016/j.celrep.2018.03.125 29694886 PMC5946722

[35] Ryndak M, Wang S, Smith I. PhoP, a key player in Mycobacterium tuberculosis virulence. Trends Microbiol. 2008;16(11):528–34. doi: 10.1016/j.tim.2008.08.006 18835713

[36] Rogers GL, Suzuki M, Zolotukhin I, Markusic DM, Morel LM, Lee B, et al. Unique Roles of TLR9- and MyD88-Dependent and -Independent Pathways in Adaptive Immune Responses to AAV-Mediated Gene Transfer. J Innate Immun. 2015;7(3):302–14. 25612611 10.1159/000369273 PMC4417066

[37] Landrigan A, Wong MT, Utz PJ. CpG and non-CpG oligodeoxynucleotides directly costimulate mouse and human CD4+ T cells through a TLR9- and MyD88-independent mechanism. J Immunol. 2011;187(6):3033–43. doi: 10.4049/jimmunol.1003414 21844387 PMC3169723

[38] Stamm CE, Collins AC, Shiloh MU. Sensing of Mycobacterium tuberculosis and consequences to both host and bacillus. Immunol Rev. 2015;264(1):204–19. doi: 10.1111/imr.12263 25703561 PMC4339209

[39] Harishankar M, Selvaraj P, Bethunaickan R. Influence of Genetic Polymorphism Towards Pulmonary Tuberculosis Susceptibility. Frontiers in Medicine. 2018;5. 10.3389/fmed.2018.00213 PMC6106802 30167433

[40] Fremond CM, Togbe D, Doz E, Rose S, Vasseur V, Maillet I, et al. IL-1 receptor-mediated signal is an essential component of MyD88-dependent innate response to Mycobacterium tuberculosis infection. J Immunol. 2007;179(2):1178–89. doi: 10.4049/jimmunol.179.2.1178 17617611

[41] Sellis D, Callahan BJ, Petrov DA, Messer PW. Heterozygote advantage as a natural consequence of adaptation in diploids. Proc Natl Acad Sci U S A. 2011;108(51):20666–71. doi: 10.1073/pnas.1114573108 22143780 PMC3251125

[42] Dallenga T, Schaible UE. Neutrophils in tuberculosis—first line of defence or booster of disease and targets for host-directed therapy? Pathog Dis. 2016;74(3). doi: 10.1093/femspd/ftw012 26903072

[43] Yeremeev V, Linge I, Kondratieva T, Apt A. Neutrophils exacerbate tuberculosis infection in genetically susceptible mice. Tuberculosis (Edinb). 2015;95(4):447–51. doi: 10.1016/j.tube.2015.03.007 25935122

[44] Fonseca KL, Maceiras AR, Matos R, Simoes-Costa L, Sousa J, Cá B, et al. Deficiency in the glycosyltransferase Gcnt1 increases susceptibility to tuberculosis through a mechanism involving neutrophils. Mucosal Immunol. 2020;13(5):836–48. doi: 10.1038/s41385-020-0277-7 32203062 PMC7434595

[45] Nair S, Huynh JP, Lampropoulou V, Loginicheva E, Esaulova E, Gounder AP, et al. Irg1 expression in myeloid cells prevents immunopathology during M. tuberculosis infection. J Exp Med. 2018;215(4):1035–45. doi: 10.1084/jem.20180118 29511063 PMC5881474

[46] Chen Z, Wang W, Liang J, Wang J, Feng S, Zhang G. Association between toll-like receptors 9 (TLR9) gene polymorphism and risk of pulmonary tuberculosis: meta-analysis. BMC Pulm Med. 2015;15:57. doi: 10.1186/s12890-015-0049-4 25948535 PMC4460768

[47] Knight M, Braverman J, Asfaha K, Gronert K, Stanley S. Lipid droplet formation in Mycobacterium tuberculosis infected macrophages requires IFN-γ/HIF-1α signaling and supports host defense. PLoS Pathog. 2018;14(1):e1006874. 29370315 10.1371/journal.ppat.1006874 PMC5800697

[48] Ní Cheallaigh C, Sheedy FJ, Harris J, Muñoz-Wolf N, Lee J, West K, et al. A Common Variant in the Adaptor Mal Regulates Interferon Gamma Signaling. Immunity. 2016;44(2):368–79. doi: 10.1016/j.immuni.2016.01.019 26885859 PMC4760121

[49] Christophe T, Jackson M, Jeon HK, Fenistein D, Contreras-Dominguez M, Kim J, et al. High content screening identifies decaprenyl-phosphoribose 2’ epimerase as a target for intracellular antimycobacterial inhibitors. PLoS Pathog. 2009;5(10):e1000645. doi: 10.1371/journal.ppat.1000645 19876393 PMC2763345

[50] Deboosère N, Iantomasi R, Queval CJ, Song OR, Deloison G, Jouny S, et al. LppM impact on the colonization of macrophages by Mycobacterium tuberculosis. Cell Microbiol. 2017;19(1). doi: 10.1111/cmi.12619 27220037 PMC5217060

[51] Raze D, Verwaerde C, Deloison G, Werkmeister E, Coupin B, Loyens M, et al. Heparin-Binding Hemagglutinin Adhesin (HBHA) Is Involved in Intracytosolic Lipid Inclusions Formation in Mycobacteria. Front Microbiol. 2018;9:2258. doi: 10.3389/fmicb.2018.02258 30333800 PMC6176652

[52] Machelart A, Salzano G, Li X, Demars A, Debrie AS, Menendez-Miranda M, et al. Intrinsic Antibacterial Activity of Nanoparticles Made of β-Cyclodextrins Potentiates Their Effect as Drug Nanocarriers against Tuberculosis. ACS Nano. 2019;13(4):3992–4007. 30822386 10.1021/acsnano.8b07902 PMC6718168

[53] Hanot Mambres D, Machelart A, Potemberg G, De Trez C, Ryffel B, Letesson JJ, et al. Identification of Immune Effectors Essential to the Control of Primary and Secondary Intranasal Infection with Brucella melitensis in Mice. J Immunol. 2016;196(9):3780–93. doi: 10.4049/jimmunol.1502265 27036913

[54] Doyle T, Moncorgé O, Bonaventure B, Pollpeter D, Lussignol M, Tauziet M, et al. The interferon-inducible isoform of NCOA7 inhibits endosome-mediated viral entry. Nat Microbiol. 2018;3(12):1369–76. doi: 10.1038/s41564-018-0273-9 30478388 PMC6329445

