# Supplementary figures and images for: Tirap controls Mycobacterium tuberculosis phagosomal acidification
Source: PLoS Pathog. 2023 Mar 8;19(3):e1011192. doi: 10.1371/journal.ppat.1011192 (PMC9994722; doi:10.1371/journal.ppat.1011192)

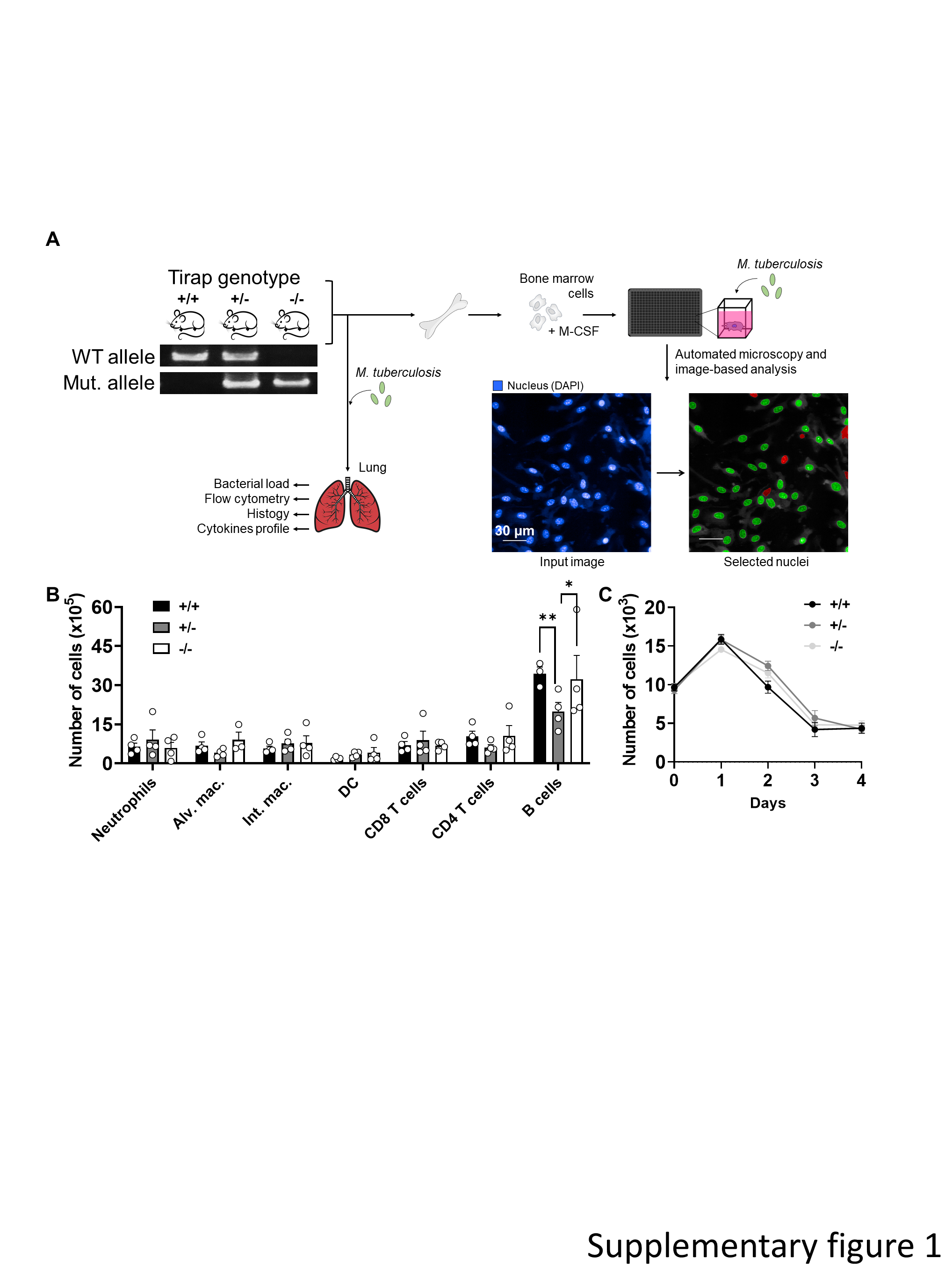

Supplement: S1 Fig — (A) Workflow of mycobacterial in vivo and in vitro infection experiments indicating different read-outs. (B) Comparison of the number of resident immune cells in the lungs of 4 naive mice for each mouse genotype. Cell numbers were normalized to the total cell number analyzed in each sample and were extrapolated to the whole lung. Shown are mean ± SEM of cells obtained in each group. (C) Comparison of BMDMs proliferation from the three mouse genotypes. * P value < 0.05, ** P value < 0.01, as determined by one-way ANOVA test. (TIF) [file ppat.1011192.s001.TIF]

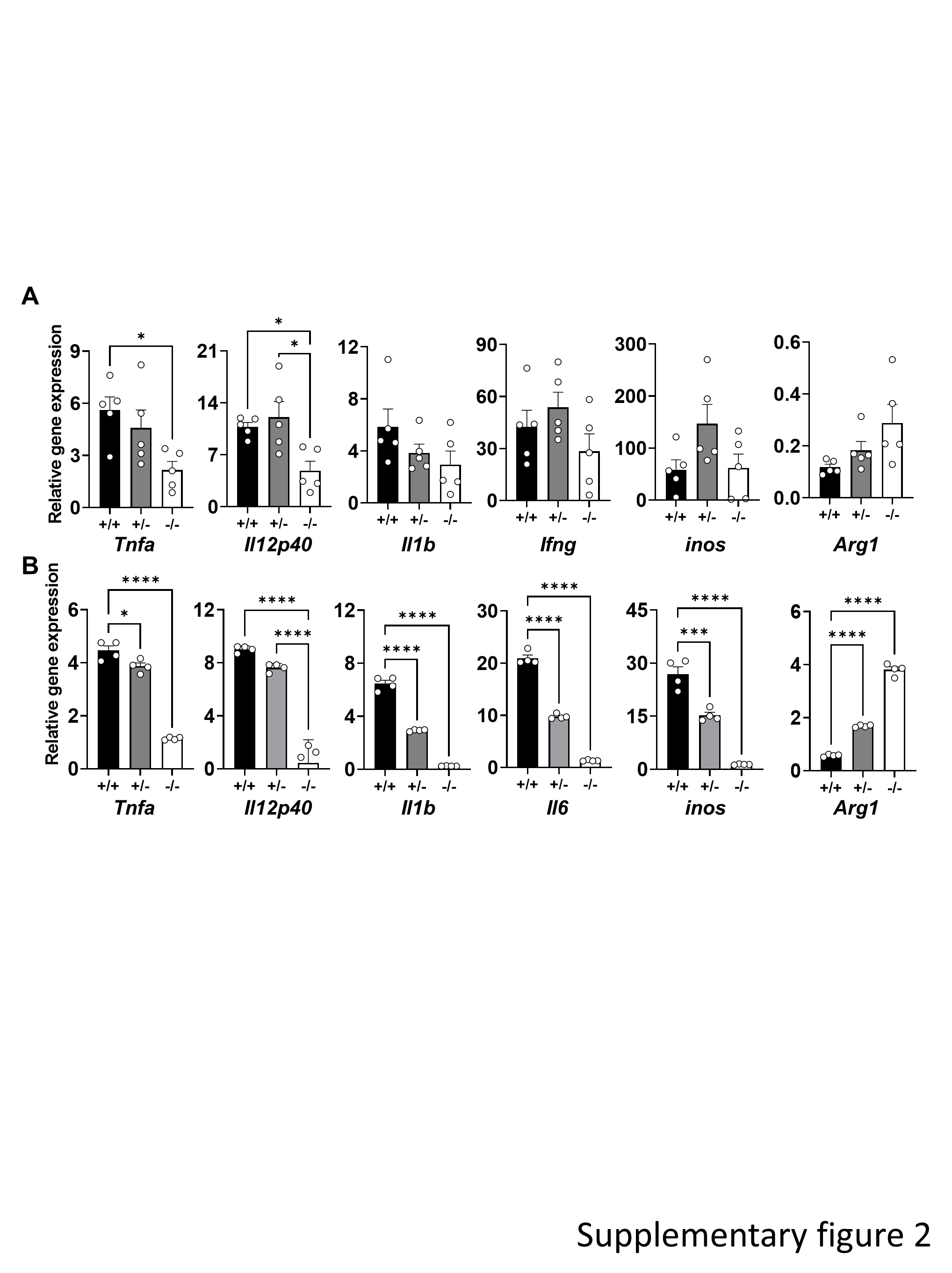

Supplement: S2 Fig — (A) Histograms showing fold increase in different cytokine expression in lungs of infected +/+, +/- and -/- mice. Shown are mean ± SEM of 5 infected mice per condition. (B) Histograms showing mean ± SEM of fold increase in different cytokine expression in BMDMs from +/+, +/- and -/- mice. Results are shown from one representative out of two independent experiments. (TIF) [file ppat.1011192.s002.TIF]

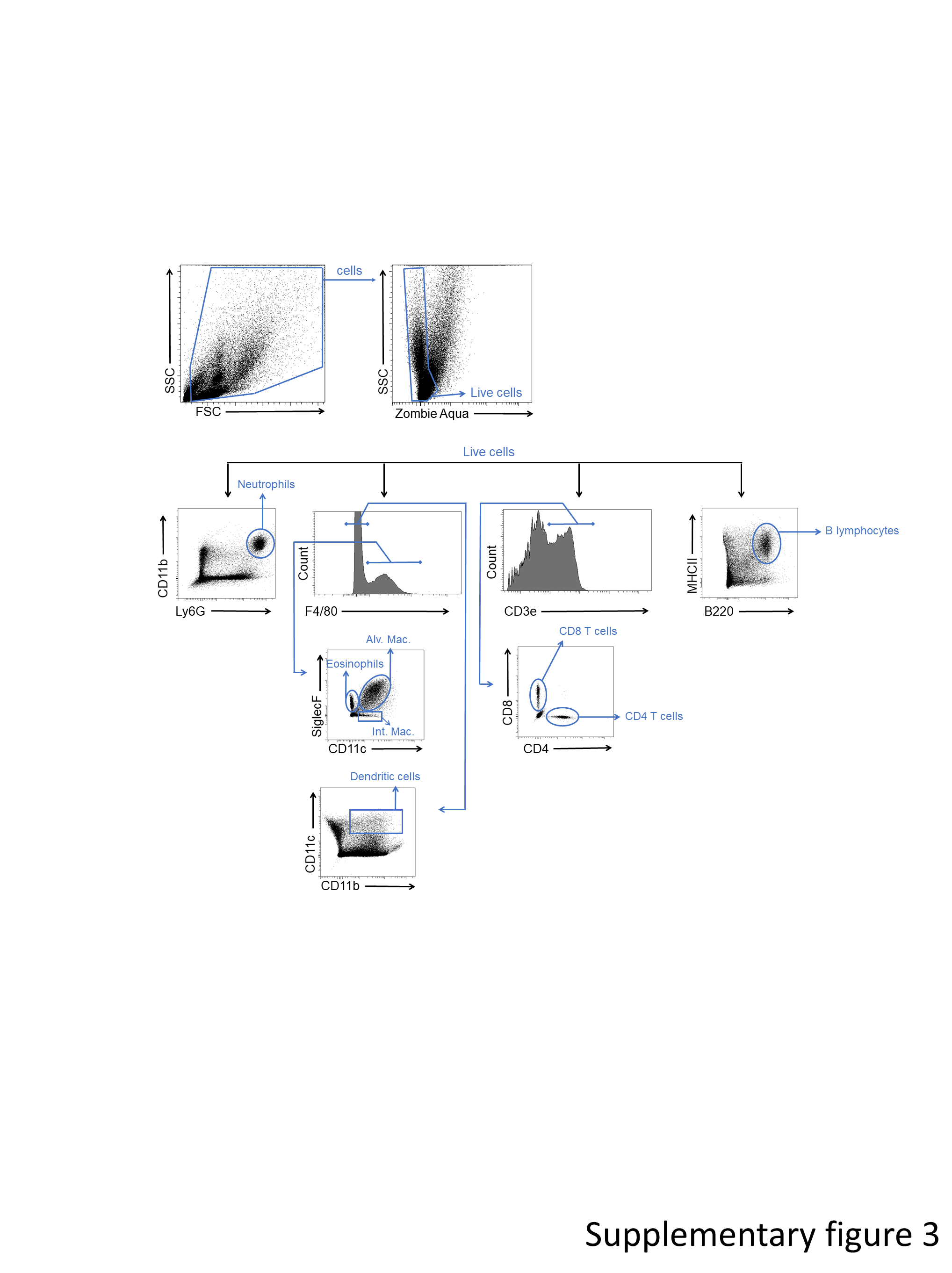

Supplement: S3 Fig — (TIF) [file ppat.1011192.s003.TIF]
